# Evaluating the implementation and impact of harm reduction vending machines in veterans supportive housing settings: a mixed-methods study protocol

**DOI:** 10.1186/s12954-025-01385-8

**Published:** 2026-01-04

**Authors:** Tessa Rife-Pennington, Michael P. Douglas, Nikki Kalani Apana, Sree Sinha, David L. Pennington

**Affiliations:** 1 San Francisco Veterans Affairs Health Care System, 4150 Clement St, San Francisco, CA 94121 USA; 2 School of Pharmacy, University of California, 490 Illinois St, 3rd floor, San Francisco, CA 94143 USA; 3 Thomas J. Long School of Pharmacy, University of the Pacific, San Francisco, CA USA; 4 School of Medicine, University of California, 533 Parnassus Ave, San Francisco, CA 94143 USA; 5 Melantha Health Psychology Consulting, PC, 1559 Sloat blvd ste B PMB 174, San Francisco, CA 94132 USA

**Keywords:** Harm reduction, Naloxone, Overdose prevention, Implementation science, HIV prevention, Veterans

## Abstract

**Background:**

Lack of access to sterile supplies among people who use drugs contributes to increased rates of infectious disease transmission, including human immunodeficiency virus, hepatitis C virus, and sexually transmitted infections. People residing in California, United States Veterans, and those who have experienced homelessness are disproportionately impacted. Syringe services programs (SSPs) are vital to reducing these harms, but access may be limited by hours of operation, geographic barriers, need for in-person interaction, and stigma. Harm reduction vending machines (HRVMs) which often dispense sterile syringes and condoms are an evidence-based strategy to increase access; however, no studies have evaluated implementation or impacts among these populations. This cross-sectional, mixed-methods study aims to evaluate the first HRVM program designed for Veterans in California who experienced homelessness and reside in supportive housing buildings.

**Methods:**

We will recruit 40 Veteran residents and 20 staff (Veterans Affairs [VA] and housing staff) at six housing buildings with a co-located HRVM. Participants will provide informed consent, complete a standardized electronic questionnaire, semi-structured qualitative interview, and be compensated via Visa gift cards ($90 for Veterans; $60 for staff). Interview transcripts will be analyzed thematically using inductive coding. Program-level data will be collected from enrollment logs, facility records, and HRVM software to evaluate reach, effectiveness, adoption, implementation, and maintenance (RE-AIM).

**Discussion:**

Findings will provide essential evidence on how HRVMs may reduce longstanding access barriers and expand delivery of life-saving harm reduction supplies to underserved Veterans. This study is the first to evaluate HRVMs in Veterans supportive housing and among this population disproportionately affected by substance use, stigma, and homelessness. Results may inform the expansion of community-based and VA SSPs nationwide. Study strengths include a theory-informed design, real-world implementation data, and attention to user and staff experiences. Limitations include reliance on self-report data, lack of a control group, and limited generalizability beyond Veterans. Future research may examine long-term health outcomes, cost-effectiveness, and feasibility of HRVMs scaled up in diverse settings. Findings from this study may guide policymakers and public health practitioners in integrating HRVMs into broader harm reduction strategies to prevent overdose, infections, and other adverse outcomes.

**Supplementary Information:**

The online version contains supplementary material available at 10.1186/s12954-025-01385-8.

## Background

In the United States (US), homelessness remains a persistent public health challenge, disproportionately impacting Veterans. California accounts for nearly one-third of all Veterans experiencing homelessness nationwide, and more than half of all unsheltered Veterans reside in the state [1]. Between 2007 and 2022, overall homelessness in California rose by 23%, and chronic homelessness increased over 43% [1]. In San Francisco alone, more than 600 Veterans were counted as homeless in 2022, reflecting the combined effects of job loss, eviction incarceration, substance use, and mental health conditions [1–4]. 

Veterans who experience homelessness frequently report substance use and psychiatric disorders as both causes and consequences of housing instability [3, 4]. Substance use disorders (SUDs) impact approximately 13% of Veterans receiving care through the Veterans Health Administration (VHA) and are associated with elevated rates of overdose and infectious disease [5, 6]. Among Veterans experiencing homelessness, co-occurring alcohol and drug use disorders are common, and return to use remains high even after housing is attained [7]. 

The convergence of substance use, unstable housing, and limited healthcare access has led to rising rates of overdose and preventable infections in California. In 2021, nearly 58,000 emergency department visits and 11,000 deaths were attributed to drug overdoses state-wide [8]. People experiencing homelessness face markedly higher infection risks, including human immunodeficiency virus (HIV), hepatitis C virus (HCV), sexually transmitted infections (STIs), and tuberculosis—in part due to syringe sharing, limited access to sterile supplies, and reduced engagement in/access to preventive services [9–13]. Veterans are disproportionately impacted; national VHA data indicate that Veterans experiencing homelessness have higher rates of HIV and viral hepatitis compared to stably housed peers [14]. 

The terms “homeless”, “unsheltered”, and “formerly homeless” Veterans describe overlapping but distinct populations. According to the US Department of Housing and Urban Development (HUD), “unsheltered homelessness” refers to individuals living in places not meant for habitation (e.g., streets, cars, parks, abandoned buildings), while “sheltered homelessness” includes those residing temporarily in emergency shelters or transitional housing [15]. In contrast, “formerly homeless” Veterans are those who have transitioned into permanent supportive housing, such as HUD-Veterans Affairs Supportive Housing (HUD-VASH) programs, which pair long-term rental assistance with case management and clinical services [16]. Although these Veterans are no longer experiencing literal homelessness, many continue to face persistent risks—stigma, poverty, and ongoing substance use or mental health challenges—that perpetuate inequities [7]. 

Harm reduction strategies, including syringe services programs (SSPs), are evidence-based approaches which provide life-saving education and resources to people who use drugs [17, 18]. Through provision of services such as sterile syringes, overdose education, naloxone, and care for HIV, HCV, STIs, and SUDs, these programs reduce rates of overdose deaths and infections, while also increasing treatment engagement [17, 18]. Despite over 30 years of evidence, SSPs within the Veterans Health Administration (VHA) only began in 2017, and many barriers limit engagement [17, 18]. For example, to receive services, Veterans must engage with a healthcare clinician during regular business hours to obtain information and resources [18]. This limits anonymity, confidentiality, and access during nights and weekends. Some Veterans do not enroll for healthcare services, and many may be ineligible due to exceeding income thresholds, dishonorable or other than honorable discharge from service, or lack of service connected conditions [19]. This limits access to VA-issued naloxone, which requires healthcare eligibility and a prescription. Furthermore, stigma and discrimination towards people who use drugs is common in healthcare settings and can have detrimental impacts on health outcomes [20–23]. 

The San Francisco Veterans Affairs Health Care System (SFVAHCS) launched its Harm Reduction and SSP in 2019 and provides services to Veterans at the San Francisco VA Medical Center (SFVAMC) and its nine outpatient clinics located in downtown San Francisco, Oakland, San Bruno, Santa Rosa, Ukiah, Clearlake, and Eureka [18]. To address access gaps, the program offers in-person outreach to formerly homeless Veterans who reside in San Francisco Bay Area supportive housing. This has been essential for increased access to harm reduction services, as Veterans are often not engaged in care, unable to come into clinic, and/or do not have a working phone. However, challenges remain due to limited staffing and ability to deliver harm reduction supplies to housing sites in a meaningful way that meets Veteran needs. Supportive housing facilities often have limited/no parking or on-site, lack of secure storage space, challenges with mail-based delivery (e.g., items damaged in transit, packages returned to sender/lost/stolen, cost for shipping), and limited public transit options. Other access barriers include service availability only during regular business hours and requiring interaction with a healthcare team member [24–27]. 

Utilization of vending machines which dispense harm reduction supplies, such as sterile syringes and naloxone, is a strategy that complements traditional SSP service delivery and further reduces access barriers [28–35]. Harm reduction vending machines (HRVMs) promote increased anonymity, acceptance, accessibility, and convenience of SSP services [30, 32, 34]. Participants who may not access traditional SSPs due to stigma, limited hours of operation, and geographic and financial barriers are more likely to engage with HRVMs [30, 32, 34, 35]. Access is also increased among people who inject drugs, have HIV, and are not engaged in treatment [32, 35]. During the COVID-19 pandemic, implementation of a HRVM was associated with higher rates of participant engagement and supply distribution compared to traditional SSP delivery methods [29]. Importantly, reductions in opioid overdose deaths and HIV incidence were also demonstrated [28, 29]. HRVMs help reduce syringe sharing, are likely to be cost efficient due to low staffing requirements, and do not lead to increased unsafe disposal of used syringes, community drug use, or vandalism [31, 33]. Key contributors to successful deployment of HRVMs include strategic geographic placement, proper maintenance, offering free supplies, and access outside of regular business hours [30, 33, 34]. 

In 2023, the SFVAHCS Harm Reduction and SSP became the first VHA site to deploy HRVMs, installing 15 across clinical and housing settings (7 in VA outpatient clinics, 2 at the SFVAMC, 6 in San Francisco Bay Area HUD-VASH housing facilities where Veterans live) [36]. To date, no programs have examined implementation strategies and impacts of harm reduction services delivery through HRVMs among Veterans and in housing settings.

## Methods

### Study aims and objectives

Our study aims to bridge this gap by evaluating the first HRVM program designed for formerly homeless Veterans who reside in San Francisco Bay Area permanent supportive housing (Table 1).

**Table 1:** Study aims and hypotheses [20, 37–46]

Aim		Hypothesis
Aim 1–reach	We seek to *evaluate* the proportion of Veteran residents who register for access to HRVMs co-located at San Francisco Bay Area HUD-VASH housing facilities and *characterize* Veterans who do and do not access supplies. We will also explore Veteran and staff feedback on registering for HRVM access and accessing harm reduction supplies.	We anticipate at least 80% of Veteran residents will register for HRVM access and that those who access supplies will report higher rates of past 30-day use of unregulated drugs, overdose in the past 6 months, and lifetime history of HIV, HCV, and STIs.
Aim 2–effectiveness	We seek to *determine* if access to HRVMs co-located at San Francisco Bay Area HUD-VASH housing facilities improves Veteran quality of life and among Veteran residents with past 30-day injection drug use, *evaluate* the potential cost-benefit for sterile syringes dispensed in prevention of HIV and HCV transmission.	We anticipate Veteran residents to report an overall positive impact on quality of life and to demonstrate positive net benefits for HRVM-dispensed sterile syringes to Veteran residents with past 30-day injection drug use.
Aim 3–adoption	We seek to *understand* barriers and facilitators for co-location of HRVMs at San Francisco Bay Area HUD-VASH housing facilities.	We anticipate the most common barriers to be “not in my backyard” (NIMBY) ideology, stigma, and lack of physical space (due to limited space in the housing buildings). We anticipate the most common facilitators to be staff support and high rates SUDs among Veteran residents.
Aim 4–implementation, maintenance, & sustainment	We seek to *describe* the overall direct costs, utilization, and modifications needed for implementation and maintenance of the San Francisco Bay Area HUD-VASH housing facility HRVMs. We will also explore Veteran- and staff feedback on the implementation, maintenance, and sustainment process.	None

Table 1 describes the proposed study aims and hypotheses mapped onto the RE-AIM framework

HRVM: Harm reduction vending machine; HUD-VASH: Housing and urban development-veterans affairs supportive housing; HIV: Human immunodeficiency virus; HCV: Hepatitis C virus; STI: Sexually transmitted infection; SUD: Substance use disorder; RE-AIM: Reach, effectiveness, adoption, implementation, maintenance

To do this, we will collect self-report data via standardized questionnaires and semi-structured qualitative interviews with Veteran residents and housing program staff to evaluate HRVM reach, effectiveness, adoption, implementation, and maintenance (RE-AIM) [42–46]. Our study employs a cross-sectional, mixed-methods design guided by the RE-AIM Model for evaluation of individual and organizational factors which determine public health impacts of a program or policy (Table 2) [42–46].

**Table 2:** Application of the RE-AIM framework to the proposed study [42–46].

Dimensions	How operationalized in proposed study	Source of data	Level
Reach the target population	Number (%) Veteran residents who register for HRVM access, and among those, number (%) accessing supplies	Program enrollment logs, facility housing logs, machine software	Organizational
	Characteristics of Veteran residents who do and do not access supplies	Qualtrics survey	Individual
	Veteran and staff feedback on registering for HRVM access and accessing harm reduction supplies	Semi-structured qualitative interviews	Individual
Effectiveness or efficacy	Veteran- and staff-reported impact on quality of life, potential unintended (negative) outcomes, and feedback on HRVMs, locations within the housing facilities, and harm reduction supplies	Semi-structured qualitative interviews	Individual
	Cost-benefit for prevention of HIV, HCV, and STIs	Qualtrics survey, machine software	Individual, Organizational
Adoption by target staff, settings, systems, and communities	Number (%) of Veteran housing facilities which accepted a HRVM	Program staff	Organizational
	Veteran- and staff-reported barriers and facilitators to co-location of HRVMs at Veteran housing facilities	Semi-structured qualitative interviews	Individual
Implementation consistency, costs, and adaptations made during delivery	Names of vendors utilized for HRVMs and initial stocked supplies	Program staff	Organizational
	Direct costs (HRVMs, internal stocked supplies, personnel)	Program staff	Organizational
	Modifications needed to HRVM location, layout, and design	Program staff	Organizational
	Description of implementation steps	Program staff	Organizational
	Veteran and stakeholder feedback on the implementation process	Semi-structured qualitative interviews	Individual
Maintenance/ sustainment of intervention effects in individuals and settings over time	Direct costs (HRVM maintenance, replenishment supplies, personnel)	Program staff	Organizational
	Modifications needed to HRVM location, layout, and design	Program staff	Organizational
	Description of implementation steps	Program staff	Organizational
	Type, quantity, days/times of harm reduction supplies dispensed via HRVM	Machine software	Organizational
	Veteran and stakeholder feedback on the maintenance/sustainment process	Semi-structured qualitative interviews	Individual

Table 2 describes the proposed study outcomes, data source, and data level (individual or organizational) mapped onto the RE-AIM framework

RE-AIM: Reach, effectiveness, adoption, implementation, maintenance; HRVM: Harm reduction vending machine; HIV: Human immunodeficiency virus; HCV: Hepatitis C virus; STI: Sexually transmitted infection

## Study design and methods

### Timeline

The proposed study will be completed within 24 months. Months 1–3 will establish Institutional Review Board (IRB) protocols and approval. Months 4–18 will enroll 40 Veterans and 20 staff (VA and housing property staff). Participants will complete an electronic Qualtrics questionnaire (Additional Files 1 and 2) and semi-structured qualitative interview (Additional Files 3, 4, and 5). During this time, program implementation and utilization data will be tracked. Months 19–21 will be devoted to processing and analyzing data. Months 22–24 will include scientific dissemination of results and initiation of a larger-scale grant application.

### Settings

The proposed study will be conducted at the SFVAMC and 6 San Francisco Bay Area HUD-VASH housing sites with a co-located HRVM. Individual housing sites are owned and managed by two non-profit organizations; provide permanent housing; have a capacity of 66–131 units; and provide a range of on-site services (e.g., mental health care; case management; peer support; part-time nursing care; free meals; tenant council and support groups). Two sites offer housing for both Veterans and low-income families.

Research study team members will complete weekly visits to the housing sites (1–2 locations/week) to offer study participation. The research study team will also be available via telephone and video to assist with recruitment, enrollment, and collection of study measures.

### Recruitment

Research team members aim to recruit approximately 6 Veterans and 3 staff per housing site. The research study team will post Veteran recruitment fliers in HUD-VASH housing buildings with a co-located HRVM (on the front of the HRVMs; in elevators and common areas). Veterans may contact study personnel directly, and on-site staff may refer Veterans to determine participation eligibility. Staff will be recruited via email from study personnel and during announcements at team meetings. The research study will begin after all HRVMs have been operational for at least 3 months.

### Participants

To be included in the present study, Veterans must reside in a SFVAHCS HUD-VASH housing site with a co-located HRVM. Veterans both with and without access to the HRVMs will be included (those with HRVM access previously completed a one-time, optional sign-up; not all Veteran residents choose to sign-up). Staff participants will include HUD-VASH property staff and VA staff (e.g., social workers, registered nurses, physicians, nurse practitioners) who work at the housing buildings. Veterans and staff who do not meet these criteria will be excluded.

### Ethics approval and consent

This study was approved by the University of California, San Francisco (UCSF) IRB (23-39677) and the SFVAHCS Human Subjects Committee (1840329). The research study team will obtain written (Veterans) or electronic (staff) consent before beginning the study questionnaire and qualitative interview.

### Study instruments

The electronic Qualtrics questionnaire for Veterans will evaluate: sociodemographic characteristics; street drug use and practices; engagement in harm reduction services; syringe disposal practices; experiences with drug overdose and naloxone; history of HIV, HCV, and STI testing and treatment; sexual practices and self-efficacy; emergency room visits and hospitalizations; and interactions with police (Additional File 1). Questions were adapted from the Collecting Demographic Data at SSPs guide [47], Core Questionnaire [48], Drug Use Disorders Identification Test (DUDIT) [49], Risk Behavior Survey (RBS) [50], internal VHA guidance on taking a brief sexual health history, and self-efficacy scale for HIV risk behaviors [51]. The electronic Qualtrics questionnaire for staff will evaluate sociodemographic characteristics, and questions were adapted from the Collecting Demographic Data at SSPs guide [47] and Core Questionnaire [48] (Additional File 2).

Semi-structured qualitative interview domains for both Veterans and staff were mapped onto the Reach, Effectiveness, Adoption, Implementation, and Maintenance (RE-AIM) framework [42–46]. Separate interview guides will be used for Veterans with and without access to the HRVMs (Additional Files 3 and 4) and staff (Additional File 5). Questions were designed as open-ended with optional prompts and probes to allow participants to fully describe their opinions, experiences, feedback, and recommendations [52–54]. 

### Study administration and participant reimbursement

Veteran participants (*n* = 40) will meet with a research team member in person and complete an electronic Qualtrics questionnaire via iPad (~ 15 min; $30 reimbursement). Veterans will also complete a semi-structured qualitative interview, which will be audio-recorded via Zoom and transcribed (~ 60 min, $60 reimbursement). Reimbursement (up to $90 total) will be provided afterwards via physical Visa gift card.

Staff participants (*n* = 20) will meet with research team members virtually via Zoom to complete a semi-structured qualitative interview (~ 60 min), which will be audio-recorded and transcribed. Afterwards, staff participants will receive an email link to complete a brief electronic Qualtrics questionnaire (~ 5 min). Staff will be reimbursed $60 via Visa gift card, which will be provided in person or mailed.

### Data collection and storage

Interview audio recordings will be validated by research team members and independently verified using human translators from Rev (Rev.com, Austin, Texas, USA). Collected Qualtrics survey and semi-structured qualitative interview data will be secured in password-protected files on the SFVAHCS and UCSF networks, and access will be restricted to research study staff. Participants will be assigned alpha-numeric identifiers, and files with identifying information will be destroyed upon completion.

Supply dispensing data will be tracked regularly throughout the study using HRVM software (de-identified). Naloxone prescription dispensing will be tracked internally by the SSP clinical team via pre-existing mechanisms (secured, password-protected file on the SFVAHCS network).

### Outcomes

Study outcomes will evaluate key facilitators and barriers across RE-AIM [42–46] domains for implementation of HRVMs in 6 San Francisco Bay Area Veterans supportive housing buildings.

### Data analysis

Descriptive statistics will be used to evaluate participant characteristics and numeric organizational-level data. For the semi-structured qualitative interviews, an inductive coding process will be used employing a thematic coding method to examine the data from Veteran and staff [55]. The research team will assign code(s) to segments of qualitative data that can be grouped into categories and themes/concepts [55]. Research team members will independently blind-code three Veteran and two staff transcripts, resolve discrepancies and refine the code book collaboratively, and divide transcripts evenly among team members for coding. Patterns in coding, categories, and themes/concepts will be examined to draw conclusions about the data [55]. Characteristics of the supportive housing facilities, staffing, and physical HRVM locations will be used to guide differences in thematic analysis. We anticipate adequate thematic saturation with the total number of participants to be recruited [52]. 

We will calculate total direct costs for the HRVMs, harm reduction supplies, maintenance, personnel (hourly salary x time spent on intervention preparation, delivery, and training) [56, 57]. To examine the potential cost-benefit, we will calculate the direct medical costs averted, valuation of quality of life gained, and valuation of years of life gained, and we will subtract the total direct costs of the intervention [56, 57]. 

## Discussion

The proposed study represents a critical advancement in the evaluation of HRVMs as an innovative strategy to improve access for Veterans experiencing or at risk for homelessness. While prior research has demonstrated HRVM effectiveness in general populations, this is the first study to assess implementation among Veterans and within supportive housing facilities [28–35]. Our findings will contribute much-needed data on how HRVMs can be used to overcome well-documented barriers to care, including stigma, limited service hours, transportation challenges, and the benefits of anonymity.

By applying the RE-AIM framework [42–46], this study will provide a comprehensive understanding of HRVM reach, effectiveness, adoption, implementation, and maintenance in supportive housing buildings where Veterans live. These findings will inform efforts to expand access to sterile syringes, naloxone, and other harm reduction supplies in California and across the country. Importantly, this project has the potential to serve as a scalable model for integrating HRVMs into other high-need settings, including shelters, rural clinics, and non-VA community programs. The results may also support federal, state, and local policy changes to promote broader adoption of HRVM-based service delivery, contributing to reduced overdose deaths and infectious disease transmission.

### Limitations

This study has several limitations. First, the evaluation is limited to a single geographic region and a specific Veteran population, which may affect generalizability to other contexts, including non-Veteran populations or rural areas. Second, the study relies on self-reported data, which may be subject to recall and social desirability biases. Third, because the evaluation is cross-sectional, findings may describe associations rather than temporal or causal relationships. Fourth, while we aim to evaluate cost-benefit and implementation outcomes, the study may not capture longer-term health outcomes, such as impact on HIV or HCV incidence, given the 24-month timeline. Finally, participation may be influenced by differential access to technology, literacy, or trust in research staff.

### Future directions

Future research should build on this pilot by evaluating longer-term health outcomes, including changes in overdose rates, infectious disease transmission, and healthcare utilization among HRVM users. These efforts should incorporate implementation science frameworks to guide scale-up in both VHA and non-VHA settings, including rural and under-resourced communities. Comparative studies between HRVMs and traditional SSPs delivery models could also clarify which services are best suited for HRVM access versus direct staff engagement. Involving Veterans with lived and living expertise in co-design and implementation could enhance relevance, acceptability, and reach. This study serves as an initial step in a broader research agenda aimed at integrating HRVMs into public health infrastructure to expand access to harm reduction services and reduce substance use-related harms nationwide.

## Supplementary Information

Below is the link to the electronic supplementary material.

Supplementary Material 1.

Supplementary Material 2.

Supplementary Material 3.

Supplementary Material 4.

Supplementary Material 5.

## Data Availability

No datasets were generated or analysed during the current study.

## Abbreviations

COVID-19: Coronavirus disease of 2019
DUDIT: Drug use disorders identification test
HRVMs: Harm reduction vending machines
HCV: Hepatitis C virus
HIV: Human immunodeficiency virus
IRB: Institutional review board
RE-AIM: Reach, effectiveness, adoption, implementation, and maintenance
SFVAHCS: San Francisco Veterans affairs health care system
STI: Sexually transmitted infection
SUD: Substance use disorder
SSPs: Syringe services programs
US: United states
HUD-VASH: US housing and urban development-veterans affairs supporting housing
VA: Veterans affairs
VHA: Veterans health administration
