# Supplementary material for: Evaluating the implementation and impact of harm reduction vending machines in veterans supportive housing settings: a mixed-methods study protocol
Source: Harm Reduct J. 2026 Jan 4;23:21. doi: 10.1186/s12954-025-01385-8 (PMC12865966; doi:10.1186/s12954-025-01385-8)
Supplement: Supplementary file 1 — Supplementary Material 1. [file 12954_2025_1385_MOESM1_ESM.pdf]

Intro

Thank you for participating in our research study! The goal of this survey is to evaluate the efficacy and usability of providing harm reduction resources via vending machines.

To understand who, where, for what, and when the harm reduction vending machines work best for, we will ask you questions related to your **demographics, mood, substance use, and general questions about your experience using harm reduction services.**

This survey should take 20 minutes, and **your responses are completely anonymous**. **Please do not include your name** or any other identifying information in your responses. We ask that you answer every question, but you always have the option to skip a question you don't feel comfortable answering.

If you have any questions about the survey, please contact Dr. Tessa Rife-Pennington (415) 319-1193.

We appreciate your time and input!

Demographics

First, I'm going to ask you a few questions about your background.

What is your date of birth? Please enter using the following format: MM/DD/YYYY (e.g., 07/15/1982).

[ ]

What best describes your racial and/or ethnic identity? Select all that apply.

- ☐ American Indian/ Native American/ Alaskan Native
- ☐ Asian
- ☐ Black/ African American
- ☐ Hispanic/ Latinx/ Latine/ Latino/ Latina
- ☐ Middle Eastern/ North African
- ☐ Native Hawaiian/ Other Pacific Islander

- ☐ White/ Caucasian
- ☐ Don't Know
- ☐ Prefer not to answer

What is your current gender identity? Select all that apply.

- ☐ Man
- ☐ Non-binary/ Gender non-conforming
- ☐ Transgender
- ☐ Woman
- ☐ [ ] Another term best describes my gender
- ☐ Prefer not to answer

What term best describes your sexual orientation?

- ○ Straight/ Heterosexual
- ○ Gay/ Lesbian
- ○ Bisexual/ Pansexual
- ○ Queer
- ○ Prefer not to answer
- ○ [ ] Another term best describes my sexual orientation

Which of the following supportive housing locations have you lived in the past year? Select all that apply.

- ☐ Stanford Hotel, 250 Kearny St
- ☐ Veterans Commons, 150 Otis St
- ☐ Veterans Academy, 1030 Girard Rd
- ☐ Edwin M Lee Apartments, 1150 3rd St
- ☐ Maceo May Apartments, 55 Cravath St
- ☐ Colma Veterans Village, 1680 Mission Rd

In the last 6 months, have you experienced one or more unhoused nights? For example:
- Sleeping in a vehicle, park, abandoned building, bus or train station, airport, or camping ground
- Temporary living arrangements in a shelter, hotel, or motel

- ◯ Yes
- ◯ No
- ◯ Don't Know
- ◯ Prefer not to answer

Drug Consumption, frequency, location, techniques

In this section, I'm going to ask you some questions about drug use.

In the past 6 months, how often did you use street drugs, such as heroin, cocaine, or methamphetamine? This does **not** include alcohol, cannabis, tobacco, or prescription medications.

- Never
- Less often than once a month
- Every month
- Every week
- Daily or almost every day

How often do you use more than one street drug on the same occasion?

- Never
- Once a month or less often
- 2-4 times a month
- 2-3 times a week

○ 4 times a week or more often

What setting(s) do you use street drugs? Select all that apply.

- ☐ At a bar, club, concert, or other public venue
- ☐ At home or in the home of someone I know (e.g., friend, family member)
- ☐ At an overdose prevention site (safe consumption site) that monitors for overdose safety, such as The Tenderloin Center
- ☐ In a car, motor vehicle, or public transportation
- ☐ In a public restroom
- ☐ In a restroom at a social service agency that monitors for overdose safety
- ☐ Outside (e.g., park, sidewalk, alleyway)
- ☐ Other, please describe: ________

Who do you typically use street drugs with? Select all that apply.

- ☐ Alone
- ☐ With another person
- ☐ With a group of people
- ☐ Other, please describe: ________

Why do you use street drugs? What benefits do you experience? Select all that apply.

- ☐ Become intoxicated
- ☐ Cost or availability (e.g., more affordable or accessible than prescription drugs)
- ☐ Curiosity (i.e., to see what it feels like)
- ☐ Increase confidence
- ☐ Decrease boredom
- ☐ Ease aftereffects of drugs (e.g., hangover effects, dope sick/withdrawal symptoms, to “come down”, cravings or urges)
- ☐ Enhance activity or performance
- ☐ Enjoy company, connect with others, or be social
- ☐ Escape
- ☐ Feel euphoria or elated
- ☐ Feel better
- ☐ Improve or enhance effects of other drugs
- ☐ Keep going or stay awake
- ☐ Lose inhibitions
- ☐ Manage a mental health or physical condition (e.g., anxiety, depression, pain, trauma, weight loss or gain)
- ☐ Relax or sleep
- ☐ Sex work
- ☐ Stop worrying
- ☐ Stressful life events or situations (e.g., homelessness, death, loss of employment)

- [ ] [ ] Other, please describe:

What types of street drugs did you use in the past 6 months? Select all that apply.

- [ ] Buprenorphine (Suboxone, Subutex, Sublocade)
- [ ] Cocaine (coke, crack)
- [ ] Fentanyl (fetty, fenty)
- [ ] Hallucinogens (e.g., ecstasy, DMT, LSD, mescaline, peyote, psilocybin)
- [ ] Heroin
- [ ] Methadone
- [ ] Methamphetamine (crystal, speed, ice, glass, crank)
- [ ] Solvents/inhalants (e.g., gas, glue, paint thinner)
- [ ] [ ] Other, please describe:

What route(s) did you use street drugs in the past 6 months? Select all that apply.

- [ ] Injecting
- [ ] Swallowing
- [ ] Rectal Use (boofing, booting, booty bumping)
- [ ] Smoking
- [ ] Snorting/sniffing

- [ ] ______ Other, please describe:

Where do you inject drugs in your body? Select all that apply.

- [ ] Crook of the arm
- [ ] Forearm or upper arm
- [ ] Hands, feet, or legs
- [ ] Neck, groin, wrist, or face
- [ ] Under the skin (skin popping)
- [ ] Muscle (e.g., muscling, muscle popping)
- [ ] ______ Other, please describe:

In the last 6 months, how often did you inject drugs using materials (e.g., syringe, cooker) that you personally re-used, including those you cleaned with bleach?

- ○ Never
- ○ Less often than once a month
- ○ Every month
- ○ Every week
- ○ Daily or almost every day

How do you prepare street drugs before using them? Select all that apply.

- ☐ I prepare my own drugs/materials.
- ☐ Someone else prepares my drugs/materials for me.
- ☐ I prepare drugs with another person, then split the drug solution (through use of the same cooker/spoon or through front/back loading)
- ☐ [ ] Other, please describe:

In the last 6 months, how often did you street use drugs using materials (e.g., syringe, cooker, rinse water, pipe, mouthpiece) previously used by someone else?

- ○ Never
- ○ Less often than once a month
- ○ Every month
- ○ Every week
- ○ Daily or almost every day

Harm Reduction Services

In this section, I'm going to ask you some questions about access to harm reduction services.

In the last 6 months, have you accessed services at a community-based syringe services program or harm reduction program? Examples programs include SF AIDS foundation, SF Drug Users Union, 6th Street Harm Reduction Center, and GLIDE.

- ○ Yes
- ○ No
- ○ Don't Know
- ○ Prefer not to answer

Which services did you access? Select all that apply.

- ☐ Meals
- ☐ Legal services
- ☐ Naloxone (Narcan) for opioid overdose reversal
- ☐ Syringes and disposal resources
- ☐ Other safer use supplies (e.g., pipes)
- ☐ Testing/treatment for HIV, hepatitis C virus, or sexually transmitted infections
- ☐ Substance use disorder care

- ☐ Other medical care
- ☐ [ ] Other, please describe:

Syringe Disposal

In this section, I'm going to ask you some questions about disposal of used syringes.

In the last 6 months, how many times have you disposed of used syringes by leaving them in a public place? For example, in the hallway outside your apartment, on the street, on the sidewalk, in a park, or in a parking lot.

- ○ Never
- ○ Less often than once a month
- ○ Every month
- ○ Every week
- ○ Daily or almost every day

In the last 6 months, how many times have you placed used syringes in a biohazardous waste (sharps)

container?

- Never
- Less often than once a month
- Every month
- Every week
- Daily or almost every day

In the last 6 months, how many times have you picked up used syringes for safe disposal that you found in a public place? For example, in the hallway outside your apartment, on the street, on the sidewalk, in a park, or in a parking lot.

- Never
- Less often than once a month
- Every month
- Every week
- Daily or almost every day

Overdose and Naloxone Use

In this section, I'm going to ask you some questions about your history of opioid overdose and use of naloxone (Narcan).

In the last 6 months, how many times have you experienced an opioid overdose? Examples of opioids include heroin, fentanyl, oxycodone, hydrocodone, morphine, methadone, and buprenorphine. By overdose, we mean a time when you lost consciousness, and someone had to do something to bring you back.

- ○ [ ] Enter number of times (enter 0 if never)
- ○ Don't Know
- ○ Prefer not to answer

In the last 6 months, how many times did someone give you a dose of naloxone (Narcan) to reverse an opioid overdose?

- ○ [ ] number of times
- ○ Don't Know
- ○ Prefer not to answer

In the last 6 months, how many times did you receive medical care after experiencing an opioid overdose? For example, receiving care from emergency responders, emergency department, or inpatient setting.

- ○ [ ] number of times
- ○ Don't Know
- ○ Prefer not to answer

In the last 6 months, how many times have you responded to an overdose for another person? For example, called 911 or administered naloxone (Narcan)?

- ○ [ ] number of times
- ○ Don't Know
- ○ Prefer not to answer

Have you ever been trained to use naloxone (Narcan)?

- ○ Yes
- ○ No
- ○ Don't know
- ○ Prefer not to answer

Are you currently carrying one or more doses of naloxone (Narcan)?

- Yes
- No
- Don't Know
- Prefer not to answer

Hepatitis C test and status

In this section, we will ask you a few questions about your history of Hepatitis C virus testing, results, and treatment.

Have you ever been tested for Hepatitis C virus?

- Yes
- No
- Don't Know
- Prefer not to answer

Have you been tested for Hepatitis C virus in the last 6 months?

- Yes
- No
- Don't Know
- Prefer not to answer

Have you even been diagnosed with hepatitis C virus?

- Yes
- No
- Don't Know
- Prefer not to answer

Have you ever received or are you currently receiving treatment for hepatitis C virus?

- Yes
- No
- Don't Know
- Prefer not to answer

Human immunodeficiency virus (HIV) test and status

In this section, we will ask you a few questions about your history of Human Immunodeficiency Virus (HIV) testing, results, and treatment.

Have you ever been tested for HIV?

- ◯ Yes
- ◯ No
- ◯ Don't Know
- ◯ Prefer not to answer

Have you been tested for HIV in the last 6 months?

- ◯ Yes
- ◯ No
- ◯ Don't Know
- ◯ Prefer not to answer

Have you even been diagnosed with HIV?

- Yes
- No
- Don't Know
- Prefer not to answer

Have you ever received or are you currently receiving treatment for HIV?

- Yes
- No
- Don't Know
- Prefer not to answer

Self-Efficacy HIV Risk Behavior

In this section, we will ask you a few questions about your HIV risk behavior.

Please indicate how sure or unsure you are about your ability to perform the following actions, even if it is not something you have done before.

	Not at all sure 1	2	3	4	Very Sure 5
Talk about safe sex with a sexual partner	○	○	○	○	○
Buy condoms in a drug store	○	○	○	○	○
Refuse to have sex with someone you don't know very well	○	○	○	○	○
Use a condom correctly if your partner wanted to	○	○	○	○	○
Refuse to inject drugs if your friends asked you to inject with them	○	○	○	○	○
Convince a partner that they should use a condom	○	○	○	○	○
Prevent a partner from having anal sex with you	○	○	○	○	○
Ask a partner about their other sexual partners	○	○	○	○	○
Refuse to use a needle that had already been used by a friend	○	○	○	○	○

Recent Sexual Behaviors

In this section, we will ask you a few questions about your sexual risk behaviors.

Have you engaged in sexual activity with another person(s) in the past 6 months?

- ○ Yes
- ○ No

Please check all types of sex you have had in the past 6 months. Select all that apply.

- ☐ Genital (penis in the vagina)
- ☐ Anal (penis in the anus)
- ☐ Oral (mouth on penis, vulva, or anus)
- ☐ Used fingers
- ☐ Used sex toys

Above, you responded that you had genital sex (penis in the vagina) in the last 6 months, what type of genital sex did you have?

- ☐ I penetrated someone's vagina
- ☐ Someone penetrated my vagina

Above, you responded that you had anal sex (penis in the anus) in the last 6 months, what type of genital sex did you have?

- ☐ I penetrated someone's anus
- ☐ Someone penetrated my anus

Above, you responded that you had oral sex (mouth on penis, vulva, or anus) in the last 6 months, what type of genital sex did you have?

- ☐ I used my mouth on someone's vulva (going down/eating out)
- ☐ I used my mouth on someone's penis (blowjob/giving head)
- ☐ I used my mouth on someone's anus (rimming)
- ☐ Someone used their mouth on my penis
- ☐ Someone used their mouth on my vulva
- ☐ Someone used their mouth on my anus

How many different partners have you engaged with sexually in the past 6 months?

- Zero
- 1 person
- 2-3 people
- 4-5 people
- Over 5 people

When I engage in sexual activity, I typically use a condom or other barrier (e.g., dental dams, gloves, or finger cots)?

- No, I never use a condom or other barrier method.
- Yes, 10-20% of the time
- Yes, 21-50% of the time
- Yes, 51-90% of the time
- Yes, 100% of the time

Have you ever exchanged sex for money, drugs, food, or a place to stay?

- No

- Yes, in the past month
- Yes, 1-6 months ago
- Yes, over 6 months ago
- Yes, over a year ago

In the past 6 months, I have been under the influence of drugs or alcohol while sexually active?

- No
- Yes, 10-20% of the time
- Yes, 21-50% of the time
- Yes, 51-90% of the time
- Yes, 100% of the time

Sexually transmitted infections (STIs)

In this section, we will ask you a few questions about your history of testing and treatment for sexually transmitted infections (STIs), such as chlamydia, gonorrhea, syphilis, herpes, trichomonas, or human papillomavirus (HPV).

Have you ever been tested for a sexually transmitted infection (STI)?

- Yes
- No
- Don't Know
- Prefer not to answer

Have you been tested for a sexually transmitted infection (STI) in the last 6 months?

- Yes
- No
- Don't Know
- Prefer not to answer

Have you even been diagnosed with a sexually transmitted infection (STI)?

- Yes
- No
- Don't Know
- Prefer not to answer

Have you ever received or are you currently receiving treatment for a sexually transmitted infection (STI)?

- Yes
- No
- Don't Know
- Prefer not to answer

Emergency Room (ER) Visits/Hospitalizations

In this section, we will ask you a few questions about recent emergency room visits, hospitalizations, and medical diagnoses.

In the last 6 months, how many times have you been to the emergency room (ER) to access health care?

- Never
- Less often than once a month
- Every month
- Every week

- ○ Daily or almost every day

In the last 6 months, how many nights have you spent in the hospital?

- ○ [ ] number of times
- ○ Don't Know
- ○ Prefer not to answer

In the last 6 months, have you experienced any of the following drug-related health problems? Select all that apply.

- ☐ None
- ☐ Abscess, cellulitis, or soft tissue infection
- ☐ Damage to the nasal septum (perforation or tear)
- ☐ Endocarditis (an infection in your heart valve)
- ☐ Heart failure, heart attack, or stroke
- ☐ Don’t know
- ☐ Prefer not to answer

Participation in substance use treatment

In this section, we will ask you a few questions about recent participation in substance use treatment.

In the last 6 months, what types of substance use disorder care have you participated in? Select all that apply.

- ☐ None
- ☐ Support groups, such as Alcoholics Anonymous (AA) or Narcotics Anonymous (NA)
- ☐ Behavioral therapies, such as cognitive behavioral therapy or contingency management
- ☐ Intensive Outpatient Program (IOP), Drug and Alcohol Treatment Program (DAT), Substance Use Posttraumatic Stress Team (SUPT), Opioid Treatment Program (OTP)
- ☐ Medically supervised withdrawal, inpatient rehabilitation, or outpatient rehabilitation
- ☐ [ ] Other, please describe

In the last 6 months, what types of medication have you taken to reduce drug cravings/use? Select all that apply.

- ☐ None
- ☐ Medication to reduce opioid cravings and/or use, including buprenorphine (Suboxone, Subutex, Sublocade), methadone, or naltrexone (Vivitrol)

- ☐ Medication to reduce alcohol cravings and/or use, including naltrexone (Vivitrol), disulfiram (Antabuse), topiramate (Topamax), gabapentin (Neurontin), acamprosate (Campral)
- ☐ Medication to reduce nicotine cravings and/or use: nicotine patches/gum/lozenge, bupropion (Wellbutrin), varenicline (Chantix)
- ☐ [ ] Other, please describe

Arrest/ police contact/ police harassment

In this section, we will ask you a few questions about recent interactions with police.

In the last 6 months, how many times have you had direct contact with the police?

- ○ Never
- ○ Less often than once a month
- ○ Every month
- ○ Every week
- ○ Daily or almost every day

In the last 6 months, how many times have you been arrested?

- ○ Never
- ○ Less often than once a month
- ○ Every month
- ○ Every week
- ○ Daily or almost every day

In the last 6 months, how many nights have you been held overnight in jail or prison?

- ○ [ ] number of nights
- ○ Don't Know
- ○ Prefer not to answer

Thank you

Thank you for participating in our program questionnaire. We value your time and your meaningful contributions. Information learned will be used to improve our harm reduction program.

Powered by Qualtrics
