# Supplementary material for: Evaluating the implementation and impact of harm reduction vending machines in veterans supportive housing settings: a mixed-methods study protocol
Source: Harm Reduct J. 2026 Jan 4;23:21. doi: 10.1186/s12954-025-01385-8 (PMC12865966; doi:10.1186/s12954-025-01385-8)
Supplement: Supplementary file 2 — Supplementary Material 2. [file 12954_2025_1385_MOESM2_ESM.pdf]

Intro

Thank you for participating in our research study! The goal of this survey is to evaluate the efficacy and usability of providing harm reduction resources via vending machines.

To understand who, where, for what, and when the harm reduction vending machines work best for, we will ask you questions related to your demographics.

This survey should take about 5 minutes, and your responses are completely anonymous. Please do not include your name or any other identifying information in your responses. We ask that you answer every question, but you always have the option to skip a question you don't feel comfortable answering.

If you have any questions about the survey, please contact Dr. Tessa Rife-Pennington (415) 319-1193. We appreciate your time and input!

A) Demographics

1. What is your date of birth? Please enter using the following format: MM/DD/YYYY (e.g., 07/15/1982).

2. What best describes your racial and/or ethnic identity? Select all that apply.

- ☐ American Indian/ Native American/ Alaskan Native
- ☐ Asian
- ☐ Black/ African American
- ☐ Hispanic/ Latinx/ Latine/ Latino/ Latina
- ☐ Middle Eastern/ North African
- ☐ Native Hawaiian/ Other Pacific Islander
- ☐ White/ Caucasian
- ☐ Don't Know
- ☐ Prefer not to answer

3. What is your current gender identity? Select all that apply.

- ☐ Man

- ☐ Non-binary/ Gender non-conforming
- ☐ Transgender
- ☐ Woman
- ☐ ______ Another term best describes my gender
- ☐ Prefer not to answer

4. What term best describes your sexual orientation?

- ○ Straight/ Heterosexual
- ○ Gay/ Lesbian
- ○ Bisexual/ Pansexual
- ○ Queer
- ○ Prefer not to answer
- ○ ______ Another term best describes my sexual orientation

5. What is the highest level of school you have completed or the highest degree you have received?

- ○ Less than high school diploma
- ○ High school diploma or equivalent (e.g., GED)
- ○ Some college but no degree
- ○ Associate degree
- ○ Bachelor degree

- ○ Graduate degree
- ○ Prefer not to answer

6. What is your job title?

- ○ Case manager
- ○ Nurse practitioner
- ○ Peer specialist
- ○ Physician
- ○ Property director
- ○ Property manager
- ○ Property monitor
- ○ Property owner
- ○ Registered nurse
- ○ Social worker
- ○ [ ] Other, please describe:
- ○ Prefer no to answer

7. Who is your current employer?

- ○ US Department of Veterans Affairs
- ○ Swords to Plowshares
- ○ Mercy Housing

- ○ [ ] Other, please describe:
- ○ Prefer not to answer

8. Which of the following supportive housing locations have you worked and/or visited in the past year? Select all that apply.

- ☐ Stanford Hotel, 250 Kearny St
- ☐ Veterans Commons, 150 Otis St
- ☐ Veterans Academy, 1030 Girard Rd
- ☐ Edwin M Lee Apartments, 1150 3rd St
- ☐ Maceo May Apartments, 55 Cravath St
- ☐ Colma Veterans Village, 1680 Mission Rd

9. What is your annual household income (total amount each individual in your home made within the last year)?

- ○ Less than $25,000
- ○ Between $25,000-$75,000
- ○ Between $75,000-$125,000
- ○ More than $125,000
- ○ Prefer not to answer

10. How many years have you worked with people who were at risk for or have experienced homelessness?

- Less than 1 year
- 1-5 years
- 6-10 years
- >10 years
- Prefer not to answer

11. How many years have you worked with people who use unregulated drugs (e.g., cocaine, methamphetamine, heroin, fentanyl)?

- Less than 1 year
- 1-5 years
- 6-10 years
- >10 years
- Prefer not to answer

12. How many years have you worked with Veterans?

- Less than 1 year
- 1-5 years
- 6-10 years

- >10 years
- Prefer not to answer

Thank you

Thank you for participating in our program questionnaire. We value your time and your meaningful contributions. Information learned will be used to improve our harm reduction program.

Powered by Qualtrics
