# Supplementary material for: Evaluating the implementation and impact of harm reduction vending machines in veterans supportive housing settings: a mixed-methods study protocol
Source: Harm Reduct J. 2026 Jan 4;23:21. doi: 10.1186/s12954-025-01385-8 (PMC12865966; doi:10.1186/s12954-025-01385-8)
Supplement: Supplementary file 3 — Supplementary Material 3. [file 12954_2025_1385_MOESM3_ESM.pdf]

Qualitative Interview Guide on Harm Reduction Vending Machines for ***Registered Veterans***

Reach:

1. **How have you heard about the Harm Reduction Vending Machine program?**
   Probe: through supportive housing staff, VA staff, other Veterans, saw them, fliers, TV advertisements at the VA

   - **How well is the Harm Reduction Vending Machine advertised at your housing site?**
     Probe: signage on each floor, in elevators, or common areas
     Prompt: Is there adequate signage? Should we add additional throughout the building? How else would it be helpful to advertise?

2. **What feedback do you have on the registration process?**
   Prompt: Was it quick and easy or long and delayed? What can we improve?

3. **What made you interested in registering for access to the Harm Reduction Vending Machines?**
   Prompt: What about it attracted you to the program?

4. **What factors impact whether you use the Harm Reduction Vending Machine at your housing site?**
   Prompt: Are there any barriers that prevent you from using it? Things that make it easier or more convenient to use?

   - **What factors impact how often you use the Harm Reduction Vending Machine at your housing site?**
     Prompt: What would make you use it more often? Less often? Are there any barriers that prevent you from using it more often?

5. **Which items did you access most often from the Harm Reduction Vending Machine?**
   Visual aid: pictures of items in machine (**Appendix 1**)
   Prompt: Are they for personal use, to share with others who need them, or both? What did you use them for?
   Probe: sharps containers for disposal of diabetes supplies or syringes for safer drug use, to pick up used syringes in the community; syringes for diabetes or drug use; condoms for safer sex; test strips to check your/others drugs before use

Effectiveness:

6. **What feedback do you have regarding the Harm Reduction Vending Machine location at your housing site?**
   Prompt: Is it in an easy or difficult location to access? Does it have adequate privacy or need more privacy? Is the area too crowded or small?

7. **What feedback do you have regarding the Harm Reduction Vending Machine overall appearance?**
   Visual aid: picture of machine front and side (**Appendix 2**)
   Probe: graphics, contact information, business card holder, colors, logo, attractiveness, appeal

Maintenance/Sustainment:

8. **What feedback do you have on how well the Harm Reduction Vending Machine functions at your housing site?**
   Probe: Does the machine work like it is supposed to? Do the items dispense properly or get stuck? Is the bin where items fall within reach? Are the machines accessible for individuals in wheelchairs or physical disabilities?

   - **What feedback do you have on the bar code wallet cards used to access the Harm Reduction Vending Machines?**
     Visual aid: pictures of wallet card (**Appendix 3**)
     Prompt: Do they work well or need improvement? Does needing a card impede your ability to access supplies? Have you needed to obtain a new card? How often?

9. **How well is the Harm Reduction Vending Machine stocked at your housing site?**
   Prompt: Have you ever gone to the machine to obtain a particular item that was not available?

10. **What feedback do you have on the supplies contained within the Harm Reduction Vending Machine at your housing site?**
    Visual aid: pictures of items in machine (**Appendix 1**)
    Probe: brands, quality, types of items
    Prompt: Do the products work as intended? Are they durable, or do they break often?

    - **What supplies would you suggest we add or remove from the Harm Reduction Vending Machine at your housing site?**
      Visual aid: pictures of items in machine (**Appendix 1**)
      Probe: any additional harm reduction supplies needed

- **Thinking about <u>current dispensing limits</u> for items in the Harm Reduction Vending Machines, are there items you need more of, or more often?**
  <u>Visual aid</u>: dispensing limit list (**Appendix 4**).
  <u>Probe</u>: which ones, how much do you need per day or week

<u>Effectiveness:</u>

11. **How has access to the Harm Reduction Vending Machine at your housing site impacted your <u>quality of life</u>?**
    <u>Probe</u>: improved, had a negative impact, in what ways; no change

    - **How has access to the Harm Reduction Vending Machine at your housing site impacted your <u>routines</u>?**
      <u>Probe</u>: less need to go to the store to buy items, less need to go to a community-based harm reduction program; no change

12. **How has access to the Harm Reduction Vending Machine at your housing site impacted your <u>overall health</u>?**
    <u>Probe</u>: improved, had a negative impact, in what ways (e.g., reduced risk for pregnancy, infections); no change

    - **How has access to the Harm Reduction Vending Machine at your housing site impacted your <u>health behaviors</u>?**
      <u>Probe</u>: more likely to use condoms during sex, more likely to use a clean syringe for injection, more likely to drugs at home rather than in the street, more likely to share harm reduction supplies with a friend; no change

<u>Adoption:</u>

13. **Have most Veteran residents at your housing site <u>who may benefit</u> registered for access to the Harm Reduction Vending Machines?**
    - Yes
    - No
    - Unsure

    If no: **How can we <u>improve</u> access for other Veteran residents?**
    <u>Probe</u>: additional registration days/times/events

14. **How <u>well received</u> is the Harm Reduction Vending Machine at your housing site?**
    <u>Probe</u>: among staff, veterans, visitors, media/news outlets
    <u>Prompt</u>: Have you heard any specific complaints or compliments?

- **How can we <u>improve acceptance</u> of the Harm Reduction Vending Machine at your housing site?**
  <u>Probe</u>: education to staff, veterans, other residents in the building

15. **What <u>concerns</u> do you have about the Harm Reduction Vending Machine at your housing site?**
    <u>Probe</u>: triggering, increased cravings or drug use, litter, vandalism, crime

    - **What <u>specific items</u> in the Harm Reduction Vending Machine do you have concerns about?**
      <u>Visual aid</u>: pictures of items in the machine (**Appendix 1**)
      <u>Probe</u>: sterile syringes, cookers, tourniquets

<u>Implementation:</u>

16. **If we installed Harm Reduction Vending Machines in other housing sites or locations, how would you recommend we <u>involve Veterans</u> in the process?**
    <u>Probe</u>: town hall or community meeting for Veteran residents, fliers in the building, phone calls or mailers to Veteran residents
    <u>Prompt</u>: How would you like to be involved?

    - **What would you recommend as an <u>ideal location for placement</u> of Harm Reduction Vending Machines?**
      <u>Probe</u>: front lobby, outside the front entrance, parking garage, bicycle room, community room
      <u>Prompt</u>: Tell me more about why that would be an ideal location (e.g., accessibility)

    - **What locations for placement of Harm Reduction Vending Machines should we <u>avoid</u>?**
      <u>Probe</u>: front lobby, outside the front entrance, parking garage, bicycle room, community room
      <u>Prompt</u>: Tell me more about why we should avoid that location (e.g., increased stigma)

    - **<u>What additional sites</u> would you recommend we install Harm Reduction Vending Machines?**
      <u>Probe</u>: specific housing sites, residential treatment programs, VA clinics
      <u>Prompt</u>: Tell me more about why those sites have a need.

Wrap Up (Remaining Time)

"Is there anything else you would like to share with the research team at this time?
"I am going to stop recording now. Thank you for your participation in this interview."

Provide handouts/resources if requested:

- Harm Reduction Vending Machine takeaway card
- Harm Reduction Program business card
- Addiction Recovery Treatment Services pamphlet
- HIV PrEP handout
- Safer injection practices handout
- Overdose education and naloxone handout

Appendix 1. Harm Reduction Vending Machine Contents.

Body lotion 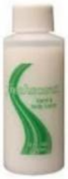	Deodorant 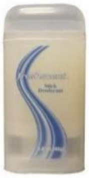	Alcohol-free mouthwash 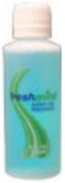	Lip balm 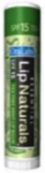
Hand sanitizer 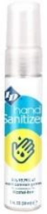	Sunscreen 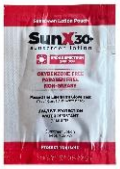	Hygiene kit 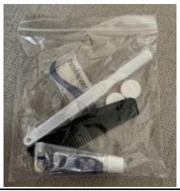	Wound care kit 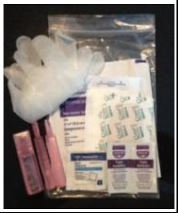
Alcohol swabs 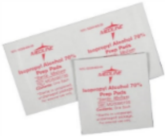	1-quart sharps container 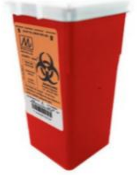	Personal sized sharps container 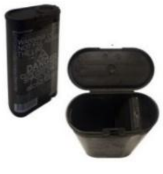	Latex-free tourniquet 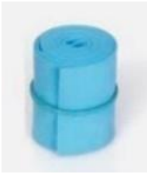
External (penile) latex condoms 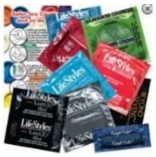	XL external (penile) latex condoms 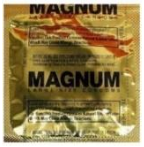	Water-based lubricant 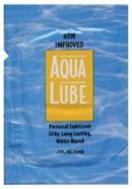	Ascorbic acid (vitamin C) powder 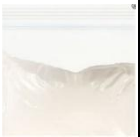
Sterile water vial. 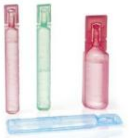	Test strips to check drugs for fentanyl 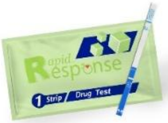	Test strips to check drugs for xylazine (AKA Tranq) 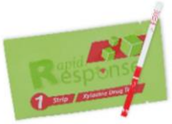	Cooker with a cotton pellet 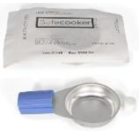
Rubber mouthpiece 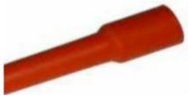	Safer snorting kit 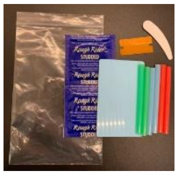	Safer rectal drug use kit (AKA boofing, booty bumping) 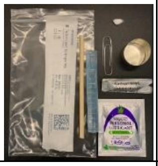	**27G** 16mm 1mL syringes 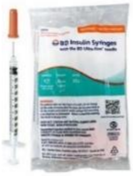
12mm 1mL syringes 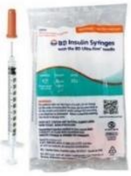	12mm 1mL syringes 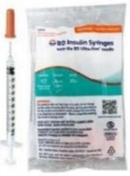	12mm 1mL syringes 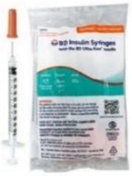	8mm 1mL syringes 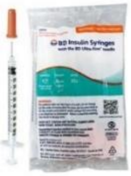

Appendix 2. Harm Reduction Vending Machine Pictures.

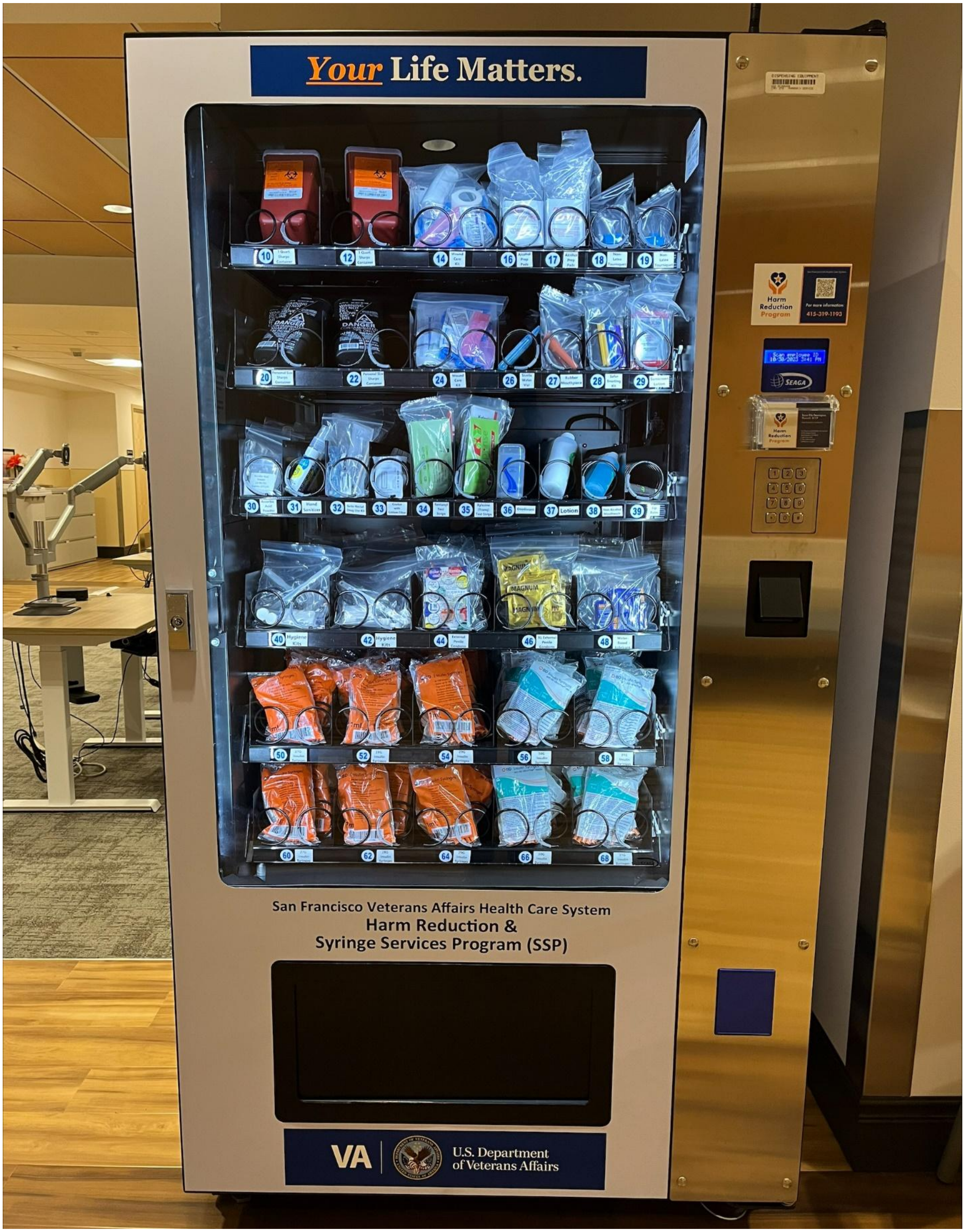

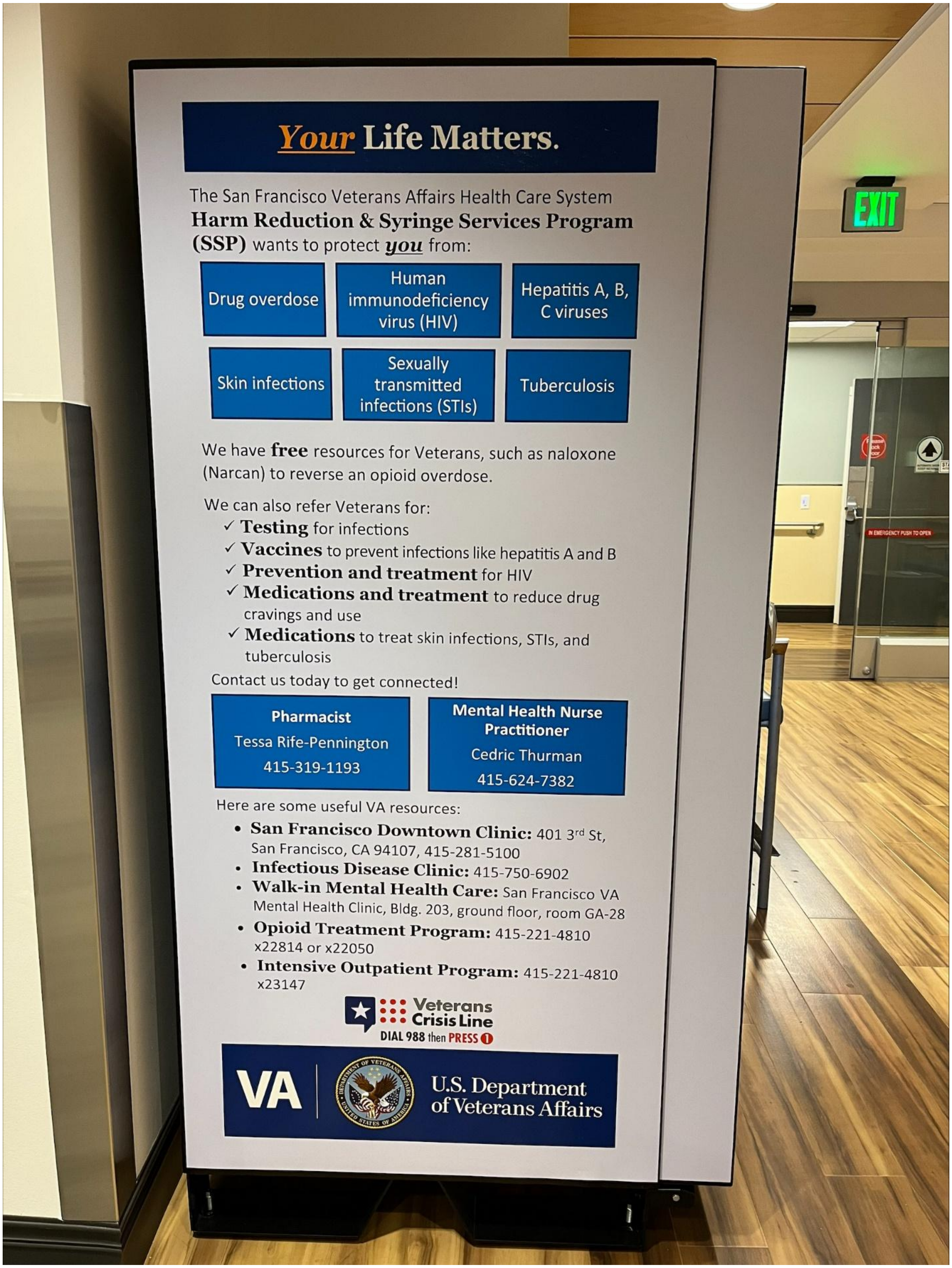

Your Life Matters.
The San Francisco Veterans Affairs Health Care System
Harm Reduction & Syringe Services Program (SSP) wants to protect you from:
Drug overdose
Human immunodeficiency virus (HIV)
Hepatitis A, B, C viruses
Skin infections
Sexually transmitted infections (STIs)
Tuberculosis
We have free resources for Veterans, such as naloxone (Narcan) to reverse an opioid overdose.
We can also refer Veterans for:
✓ Testing for infections
✓ Vaccines to prevent infections like hepatitis A and B
✓ Prevention and treatment for HIV
✓ Medications and treatment to reduce drug cravings and use
✓ Medications to treat skin infections, STIs, and tuberculosis
Contact us today to get connected!
Pharmacist
Tessa Rife-Pennington
415-319-1193
Mental Health Nurse Practitioner
Cedric Thurman
415-624-7382
Here are some useful VA resources:
• San Francisco Downtown Clinic: 401 3rd St, San Francisco, CA 94107, 415-281-5100
• Infectious Disease Clinic: 415-750-6902
• Walk-in Mental Health Care: San Francisco VA Mental Health Clinic, Bldg. 203, ground floor, room GA-28
• Opioid Treatment Program: 415-221-4810 x22814 or x22050
• Intensive Outpatient Program: 415-221-4810 x23147
Veterans Crisis Line
DIAL 988 then PRESS 1
VA
U.S. Department of Veterans Affairs
EXIT

Appendix 3. Bar Code Wallet Card

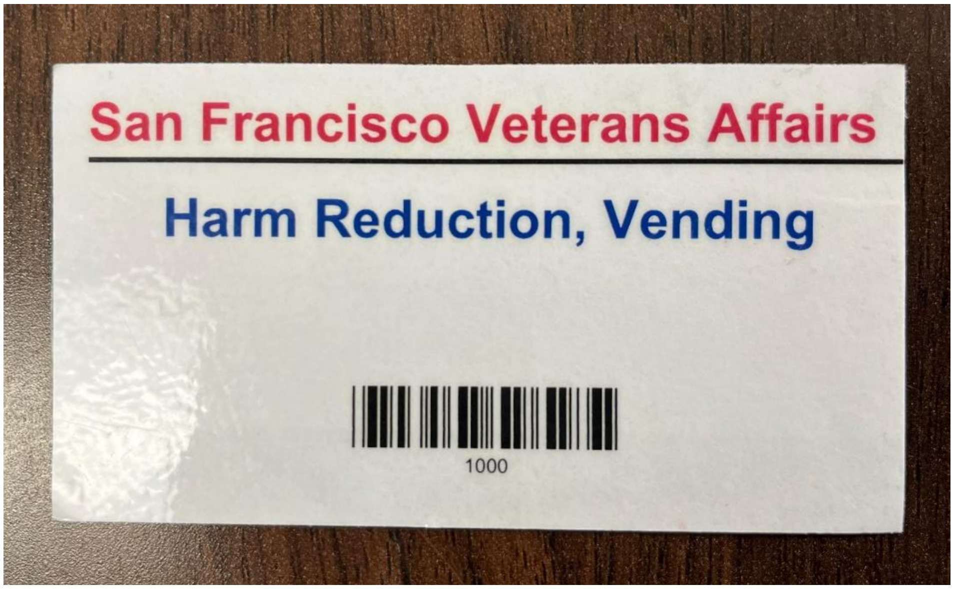

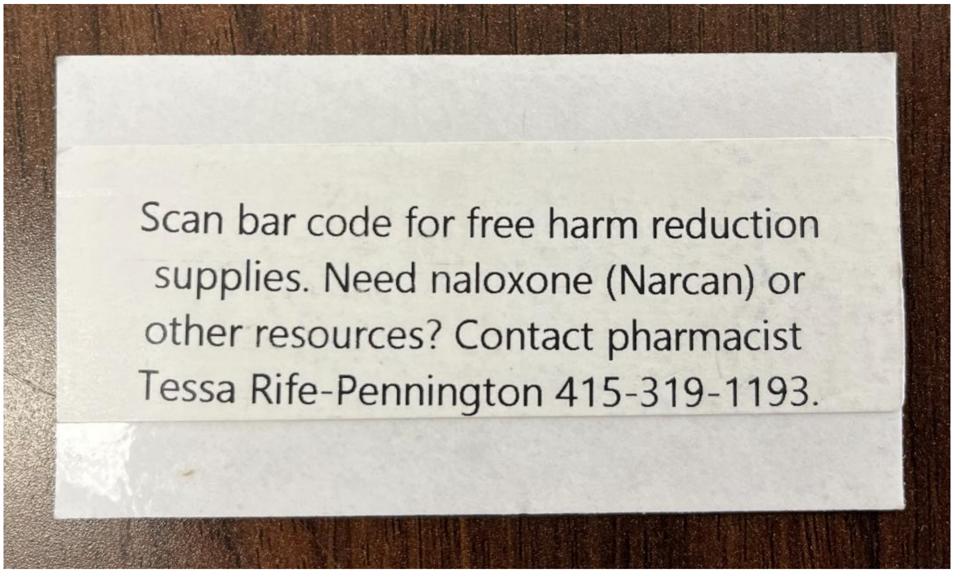

Appendix 4. Dispensing Limits

Item	Allowed Per Day
27G insulin syringe	1 bag (10 syringes)
28G insulin syringe	1 bag (10 syringes)
29G insulin syringe	1 bag (10 syringes)
30G insulin syringe	1 bag (10 syringes)
31G insulin syringe	1 bag (10 syringes)
Alcohol swabs	1 bag (10 swabs)
Ascorbic acid (vitamin C) powder	1 bag
Cooker with cotton filter	6 cookers with filters
Safer rectal use kit	2 kits
Sterile water vial	6 vials
Wound care kit	1 kit

Item	Allowed Per Week
1 quart sharps container	1 container
Alcohol-free hand sanitizer	1 bottle
Aqua lube	2 bags (20 single-use packets)
Deodorant	1 bottle
Fentanyl test strips	1 bag (10 test strips)
Hygiene kit	1 kits
Latex condoms	2 bags (20 condoms)
Lip balm	1 tube
Lotion	1 bottle
Mouthwash	1 bottle
Personal size sharps container	1 container
Rubber mouthpiece	1 mouthpiece
Xylazine test strips	1 bag (10 test strips)
XL latex condoms	2 bags (20 condoms)
Safer snorting kit	7 kits **starting 7/1/24, changed from 1/day
Sunscreen	7 bags (3 single-use packets) **starting 7/1/24, changed from 1/day
Tourniquet	7 tourniquets **starting 7/1/24, changed from 1/day
