# Supplementary material for: Evaluating the implementation and impact of harm reduction vending machines in veterans supportive housing settings: a mixed-methods study protocol
Source: Harm Reduct J. 2026 Jan 4;23:21. doi: 10.1186/s12954-025-01385-8 (PMC12865966; doi:10.1186/s12954-025-01385-8)
Supplement: Supplementary file 4 — Supplementary Material 4. [file 12954_2025_1385_MOESM4_ESM.pdf]

Qualitative Interview Guide on Harm Reduction Vending Machines for ***Non-Registered Veterans***

Reach:

1. **How have you heard about the Harm Reduction Vending Machine program?**
   Probe: through supportive housing staff, VA staff, Veterans, saw them, fliers, TV advertisements at the VA

   - **How well is the Harm Reduction Vending Machine advertised at your housing site?**
     Probe: signage on each floor, in elevators, or common areas
     Prompt: Is there adequate signage? Should we add additional throughout the building? How else would it be helpful to advertise?

2. **Why have you not registered for access to the Harm Reduction Vending Machine?**
   Probe: lack of interest, unclear registration process, concerns about the items in the machine, already have access through other means (e.g., community-based syringe services program, receive as a prescription, purchase what I need), quality of items in the machine, do not need the items in the machine

   - **What would make you interested in registering for access?**
     Probe: specific brands, quality, types of items you need or would be interested in

   - **How could we improve or increase awareness of the registration process?**

Adoption[1]:

Harm reduction refers to public health programs that reduce the harms related to drug use, without requiring people to stop using drugs. One example is providing naloxone (also called Narcan) to help save someone's life if they overdose on opioids. Another example is offering sterile syringes to people who may inject drugs to prevent infections, like hepatitis C virus and HIV.

[1] Adapted from: Wild TC, Koziel J, Anderson-Baron J, Asbridge M, Belle-Isle L, Dell C, Elliott R, Hathaway A, MacPherson D, McBride K, Pauly B, Strike C, Galovan A, Hyshka E. Public support for harm reduction: A population survey of Canadian adults. PLoS One. 2021 May 19;16(5):e0251860. doi: 10.1371/journal.pone.0251860. PMID: 34010338; PMCID: PMC8133460.

There are many different opinions about harm reduction. Many supporters think these programs can significantly reduce death and the transmission of disease among people who use drugs. Supporters also believe that these programs can bring them into contact with health and social services that could help in their recovery journey. Opponents may argue that harm reduction programs encourage crime and drug use and should not be offered.

3. **How would you describe your personal level of support for <u>harm reduction</u>:**
   <u>Probe</u>: support or do not support; feel neutral or no opinion

   - **How would you describe your personal level of support for distribution of <u>naloxone (Naloxone)</u> for opioid overdose reversal?**
     <u>Probe</u>: support or do not support; feel neutral or no opinion

   - **How would you describe your personal level of support for distribution of <u>sterile syringes</u> that may be used to inject drugs?**
     <u>Probe</u>: support or do not support; feel neutral or no opinion

4. **How comfortable would you be talking to <u>someone who uses illicit drugs</u>?**
   <u>Prompt</u>: Would you feel afraid or hesitant to talk to them? What if the person was a friend or family member? Would you feel upset, disturbed, or ashamed?

5. **How would you describe your personal level of support for the <u>harm reduction vending machine</u>?**
   <u>Probe</u>: support or do not support; feel neutral or no opinion
   <u>Prompt</u>: Do you feel that it belongs in a building where you live?

   - **What <u>concerns</u> do you have about the Harm Reduction Vending Machine at your housing site?**
     <u>Probe</u>: triggering, increased cravings or drug use, litter

   - **What <u>specific items</u> in the Harm Reduction Vending Machine do you have concerns about?**
     <u>Visual aid</u>: pictures of items in machine (**Appendix 1**)
     <u>Probe</u>: sterile syringes, cookers, tourniquets

   - **Are there <u>specific items</u> in the Harm Reduction Vending Machine that you support Veterans having access to?**
     <u>Visual aid</u>: pictures of items in machine (**Appendix 1**)
     <u>Probe</u>: deodorant, lotion, mouthwash, lip balm, sunscreen

<u>Effectiveness:</u>

6. **What feedback do you have regarding the Harm Reduction Vending Machine <u>location</u> at your housing site?**
   <u>Prompt</u>: Is it in an easy or difficult location to access? Does it have adequate privacy or need more privacy? Is the area too crowded or small?

7. **What feedback do you have regarding the Harm Reduction Vending Machine <u>overall appearance</u>?**
   <u>Visual aid</u>: picture of machine front and side (**Appendix 2**)
   <u>Probe</u>: graphics, contact information, business card holder, colors, logo, attractiveness, appeal

8. **How has having the Harm Reduction Vending Machine at your housing site impacted your <u>routines</u>?**
   <u>Probe</u>: improved, had a negative impact, in what ways; no change
   <u>Prompt</u>: Do you avoid the area where it is located?

9. **How has having the Harm Reduction Vending Machine at your housing site impacted your <u>quality of life</u>?**
   <u>Probe</u>: improved, had a negative impact, in what ways; no change

   - **How has having the Harm Reduction Vending Machine at your housing site impacted your <u>living environment</u>?**
     <u>Probe</u>: improved, had a negative impact, in what ways; no change

   - **How has having the Harm Reduction Vending Machine at your housing site impacted your <u>interactions with Veterans and staff at the housing site</u>?**
     <u>Probe</u>: improved, had a negative impact, in what ways; no change

<u>Wrap Up (Remaining Time)</u>

Is there anything else you would like to share with the research team at this time?
"I am going to stop recording now."
"Thank you for your participation in this interview."

Provide handouts/resources if requested:

- Harm Reduction Vending Machine takeaway card
- Harm Reduction Program business card
- Addiction Recovery Treatment Services pamphlet
- HIV PrEP handout
- Safer injection practices handout
- Overdose education and naloxone handout

Appendix 1. Harm Reduction Vending Machine Contents.

Body lotion 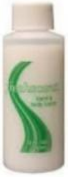	Deodorant 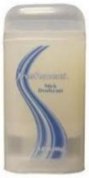	Alcohol-free mouthwash 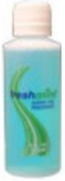	Lip balm 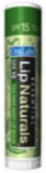
Hand sanitizer 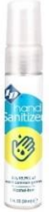	Sunscreen 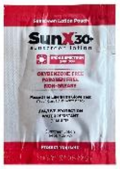	Hygiene kit 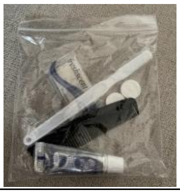	Wound care kit 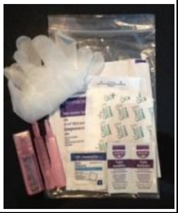
Alcohol swabs 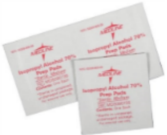	1-quart sharps container 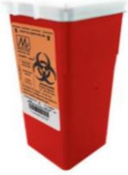	Personal sized sharps container 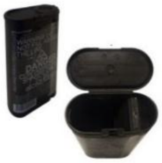	Latex-free tourniquet 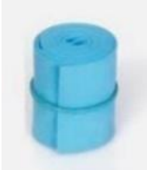
External (penile) latex condoms 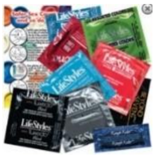	XL external (penile) latex condoms 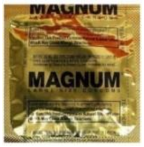	Water-based lubricant 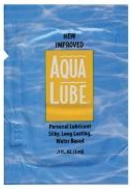	Ascorbic acid (vitamin C) powder 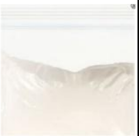
Sterile water vial. 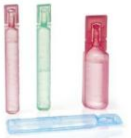	Test strips to check drugs for fentanyl 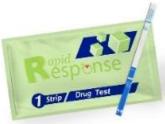	Test strips to check drugs for xylazine (AKA Tranq) 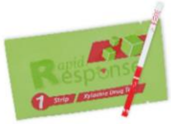	Cooker with a cotton pellet 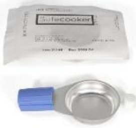
Rubber mouthpiece 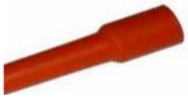	Safer snorting kit 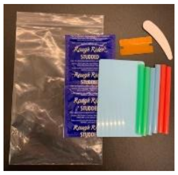	Safer rectal drug use kit (AKA boofing, booty bumping) 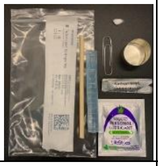	**27G** 16mm 1mL syringes 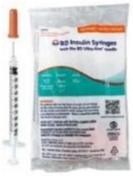
12mm 1mL syringes 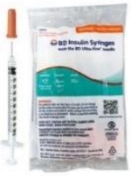	12mm 1mL syringes 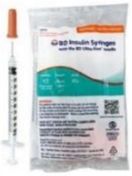	12mm 1mL syringes 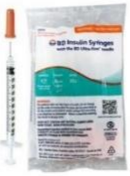	8mm 1mL syringes 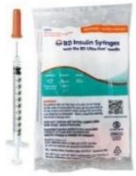

Appendix 2. Harm Reduction Vending Machine Pictures.

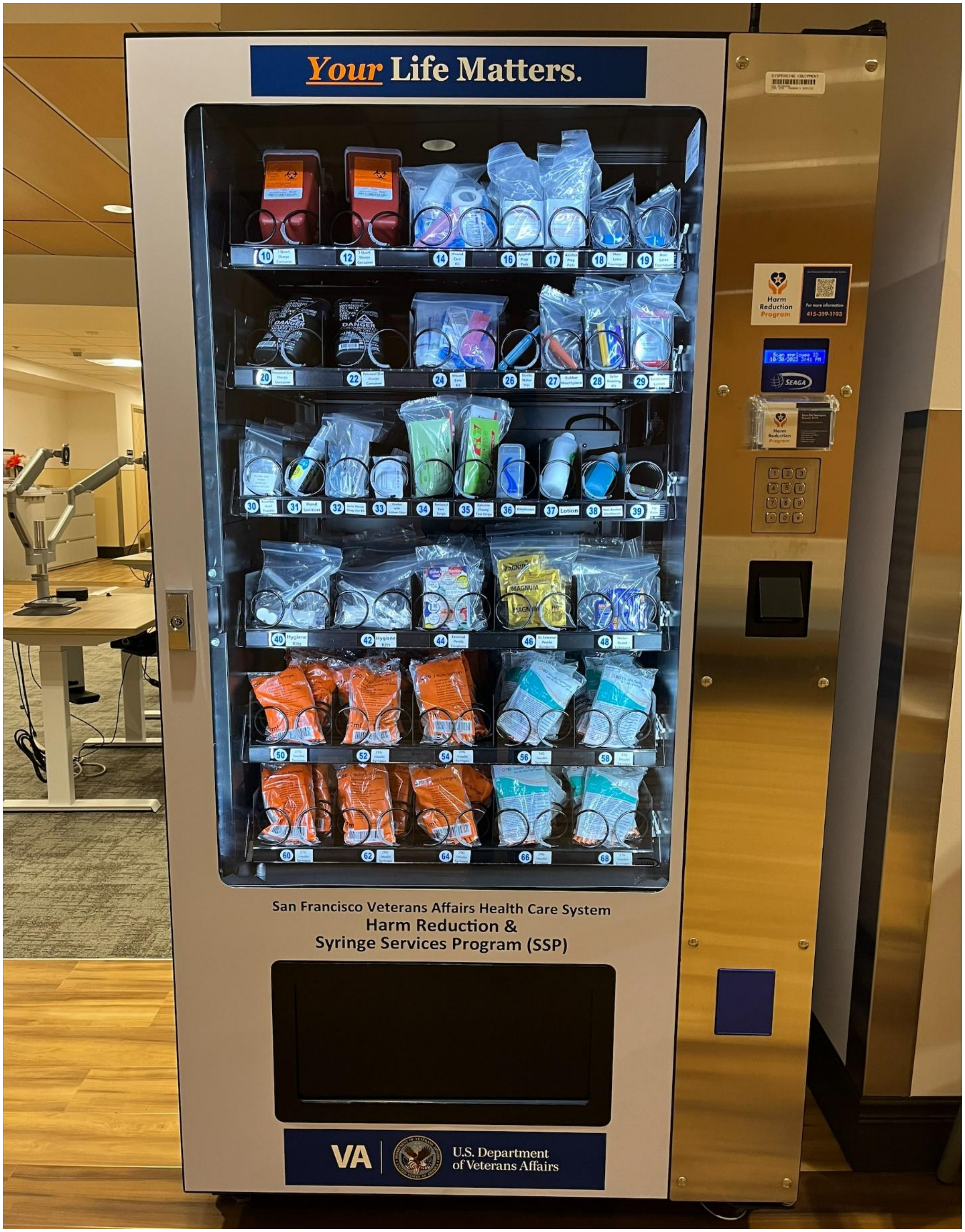

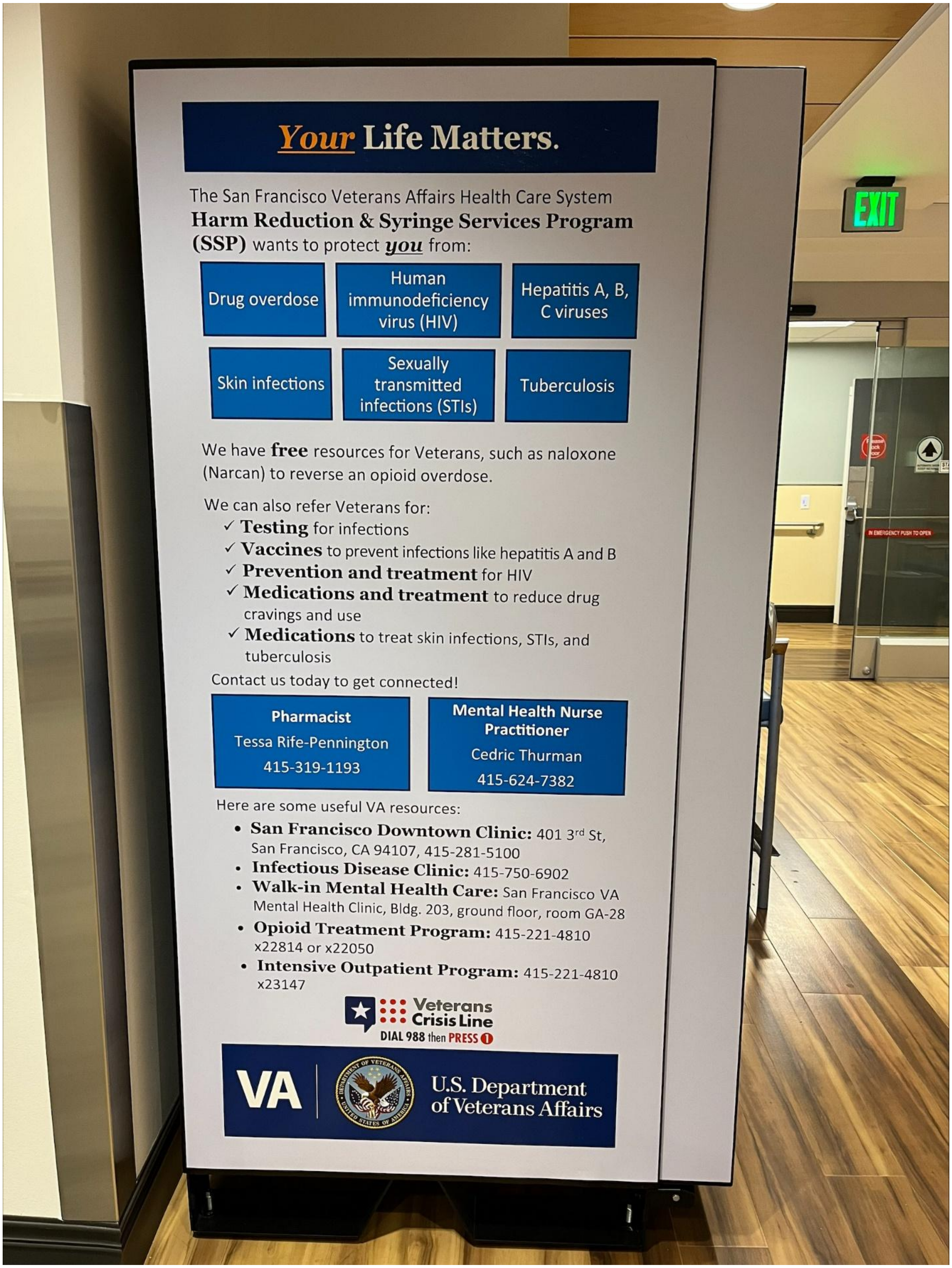
Your Life Matters.
The San Francisco Veterans Affairs Health Care System
Harm Reduction & Syringe Services Program (SSP) wants to protect you from:
Drug overdose
Human immunodeficiency virus (HIV)
Hepatitis A, B, C viruses
Skin infections
Sexually transmitted infections (STIs)
Tuberculosis
We have free resources for Veterans, such as naloxone (Narcan) to reverse an opioid overdose.
We can also refer Veterans for:
✓ Testing for infections
✓ Vaccines to prevent infections like hepatitis A and B
✓ Prevention and treatment for HIV
✓ Medications and treatment to reduce drug cravings and use
✓ Medications to treat skin infections, STIs, and tuberculosis
Contact us today to get connected!
Pharmacist
Tessa Rife-Pennington
415-319-1193
Mental Health Nurse Practitioner
Cedric Thurman
415-624-7382
Here are some useful VA resources:
• San Francisco Downtown Clinic: 401 3rd St, San Francisco, CA 94107, 415-281-5100
• Infectious Disease Clinic: 415-750-6902
• Walk-in Mental Health Care: San Francisco VA Mental Health Clinic, Bldg. 203, ground floor, room GA-28
• Opioid Treatment Program: 415-221-4810 x22814 or x22050
• Intensive Outpatient Program: 415-221-4810 x23147
Veterans Crisis Line
DIAL 988 then PRESS 1
VA
U.S. Department of Veterans Affairs
EXIT
