# Supplementary material for: Evaluating the implementation and impact of harm reduction vending machines in veterans supportive housing settings: a mixed-methods study protocol
Source: Harm Reduct J. 2026 Jan 4;23:21. doi: 10.1186/s12954-025-01385-8 (PMC12865966; doi:10.1186/s12954-025-01385-8)
Supplement: Supplementary file 5 — Supplementary Material 5. [file 12954_2025_1385_MOESM5_ESM.pdf]

Qualitative Interview Guide on Harm Reduction Vending Machines for *Staff*

Reach:

1. **How have you heard about the Harm Reduction Vending Machine program?**
   Probe: through supportive housing staff, VA staff, Veterans, saw them, fliers, TV advertisements at the VA

   - **How well is the Harm Reduction Vending Machine advertised at the housing site(s)?**
     Probe: signage on each floor, in elevators, or common areas
     Prompt: Is there adequate signage? Should we add additional throughout the building? How else would it be helpful to advertise?

2. **What feedback do you have on how Veterans register for access to the Harm Reduction Vending Machines?**
   Prompt: Is it quick and easy or long and delayed? What can we improve?

3. **Please describe your role(s) in the Harm Reduction Vending Machine program.**
   Probe: approved or helped obtain approval for installation, coordinated on-site installation, refer or register Veterans for access, provide Veteran education on the program, inform the program lead when the machines are empty or need maintenance

   - **How else would you like to be involved?**

Effectiveness:

4. **What feedback do you have on the Harm Reduction Vending Machine location(s) at the housing site(s)?**
   Prompt: Is it in an easy or difficult location to access? Does it have adequate privacy or need more privacy? Is the area too crowded or small?

5. **What feedback do you have on the Harm Reduction Vending Machine overall appearance?**
   Visual aid: picture of machine front and side (**Appendix 1**)
   Probe: graphics, contact information, business card holder, colors, logo, attractiveness, appeal

Maintenance/Sustainment:

6. **What feedback do you have on how well the Harm Reduction Vending Machine functions at the housing site(s)?**
   Prompt: Does the machine work like it is supposed to? Do the items dispense properly or get stuck? Is the bin where items fall within reach? Are the machines accessible for individuals in wheelchairs or physical disabilities?

   - **What feedback do you have on the bar code wallet cards used to access the Harm Reduction Vending Machines?**
     Visual aid: show picture of wallet card (**Appendix 2**)
     Prompt: Do they work well or need improvement? Have you had to help a Veteran obtain a new card? How often? What happened (e.g., lost, damaged)?

7. **How well is the Harm Reduction Vending Machine stocked at the housing site(s)?**
   Prompt: Is it adequately stocked or frequently empty? Which products are commonly empty?

8. **What feedback do you have on the supplies contained within the Harm Reduction Vending Machine at the housing site(s)?**
   Visual aid: pictures of items in machine (**Appendix 3**)
   Probe: brands, quality, types of items

   - **What supplies would you suggest we add or remove from the Harm Reduction Vending Machine at the housing site(s)?**
     Visual aid: pictures of items in machine (**Appendix 3**)
     Probe: any additional harm reduction supplies needed

   - **Thinking about current dispensing limits for items in the Harm Reduction Vending Machines, are there items Veterans need more of, or more often?**
     Visual aid: dispensing limit list (**Appendix 4**).
     Probe: which ones, how much per day or week

Effectiveness:

9. **How has access to the Harm Reduction Vending Machine at the housing site(s) impacted your work satisfaction?**
   Probe: improved, had a negative impact, in what ways; no change

   - **How has access to the Harm Reduction Vending Machine at the housing site(s) impacted your work duties?**
     Probe: any new work duties, change in work duties/flow, extra time needed to complete work; no change

- **How has access to the Harm Reduction Vending Machine at your housing site impacted your <u>interactions with Veterans</u>?**
  <u>Probe</u>: positive, negative, in what ways; no change

10. **How has access to the Harm Reduction Vending Machine at the housing site(s) impacted your <u>work safety</u>?**
    <u>Prompt</u>: Have you noticed an increase in litter, used syringes, or other supplies contained within the vending machine discarded throughout the housing site(s) (i.e., not in appropriate disposal receptacle)? Have you noticed an increase in vandalism or crime?

    - **How has access to the Harm Reduction Vending Machine at the housing site(s) impacted your <u>work environment</u>?**
      <u>Probe</u>: increased need to store supplies on site, space needs

<u>Adoption:</u>

11. **Have most Veteran residents at the housing site(s) <u>who may benefit</u> registered for access to the Harm Reduction Vending Machines?**
    - Yes
    - No
    - Unsure

    If no: **How can we <u>improve</u> access for other Veteran residents?**
    <u>Probe</u>: additional registration days/times/events

12. **How <u>well received</u> is the Harm Reduction Vending Machine at the housing site(s)?**
    <u>Probe</u>: among staff, veterans, visitors, media/news outlets
    <u>Prompt</u>: Have you heard any specific complaints or compliments?

    - **How can we <u>improve acceptance</u> of the Harm Reduction Vending Machine at the housing site(s)?**
      <u>Probe</u>: education to staff, veterans, other residents in the building

13. **What <u>concerns</u> do you have about the Harm Reduction Vending Machine at the housing site(s)?**
    <u>Probe</u>: triggering, increased cravings or drug use, litter, vandalism, crime

    - **What <u>specific items</u> in the Harm Reduction Vending Machine do you have concerns about?**
      <u>Visual aid</u>: pictures of items in machine (**Appendix 3**)
      Probe: safer use supplies, like sterile syringes, cookers, tourniquets

Implementation:

14. **What feedback do you have on how we installed and implemented the Harm Reduction Vending Machine at housing site(s)?**
    Prompt: What was the process like? Was there adequate communication and awareness among staff and residents?

    - **If we installed additional Harm Reduction Vending Machines at other housing sites, how could we improve the process?**
      Probe: town hall or community meeting for Veteran residents, fliers in the building, phone calls or mailers to Veteran residents

15. **What would you recommend as an ideal location for placement of Harm Reduction Vending Machines?**
    Probe: front lobby, outside the front entrance, parking garage, bicycle room, community room
    Prompt: Tell me more about why that would be an ideal location (e.g., accessibility, visibility, high or low traffic area).

    - **What locations for placement of Harm Reduction Vending Machines should we avoid?**
      Probe: front lobby, outside the front entrance, parking garage, bicycle room, community room
      Prompt: Tell me more about why we should avoid that location (e.g., accessibility, visibility, high or low traffic area).

    - **What additional sites would you recommend we install Harm Reduction Vending Machines?**
      Probe: specific housing sites, residential treatment programs, VA clinics
      Prompt: Tell me more about why those sites have a need.

Wrap Up (Remaining Time)

"Is there anything else you would like to share with the research team at this time?
"I am going to stop recording now. Thank you for your participation in this interview."

Provide handouts/resources if requested:

- Harm Reduction Vending Machine takeaway card
- Harm Reduction Program business card
- Addiction Recovery Treatment Services pamphlet
- HIV PrEP handout
- Safer injection practices handout
- Overdose education and naloxone handout

Appendix 1. Harm Reduction Vending Machine Pictures.

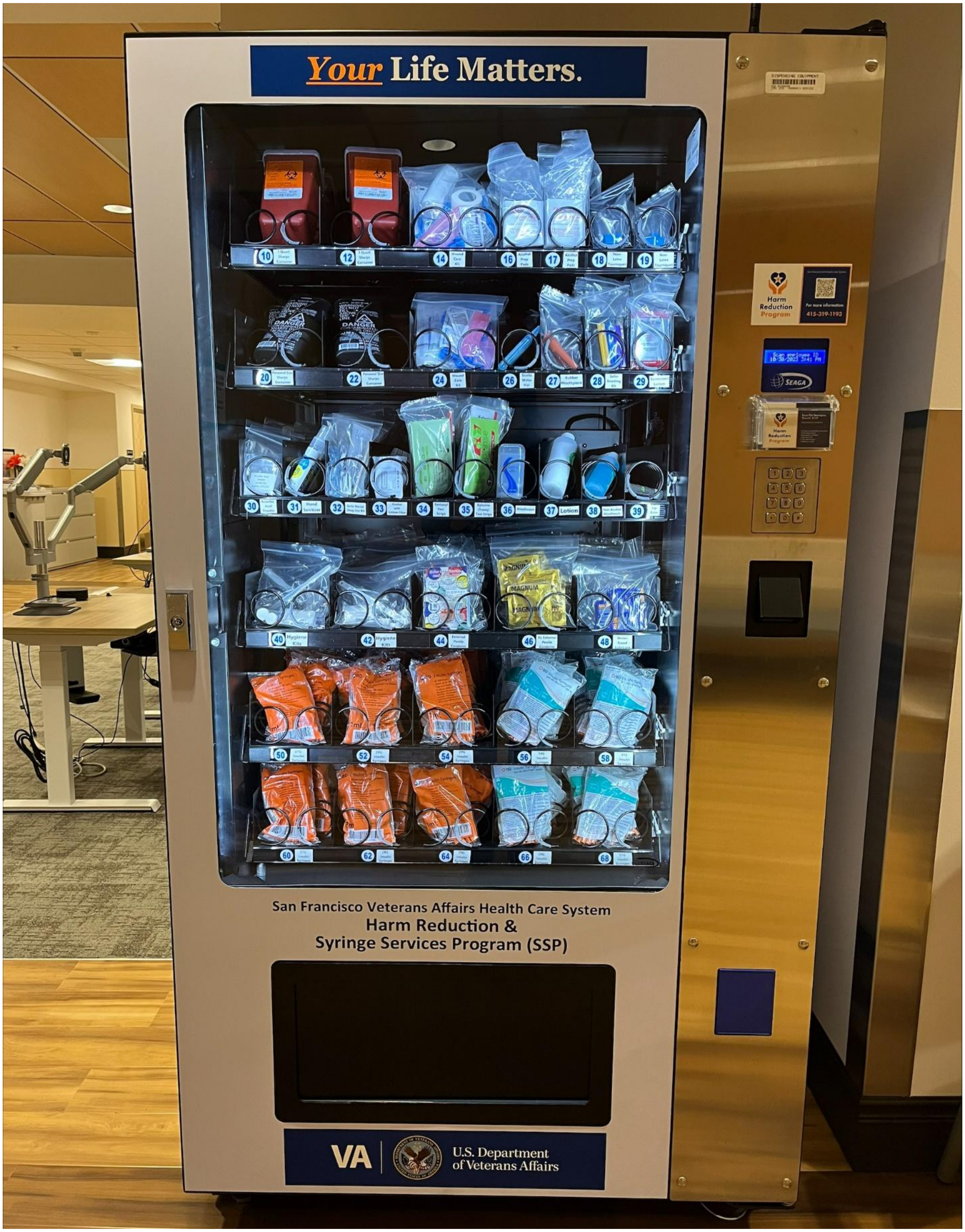

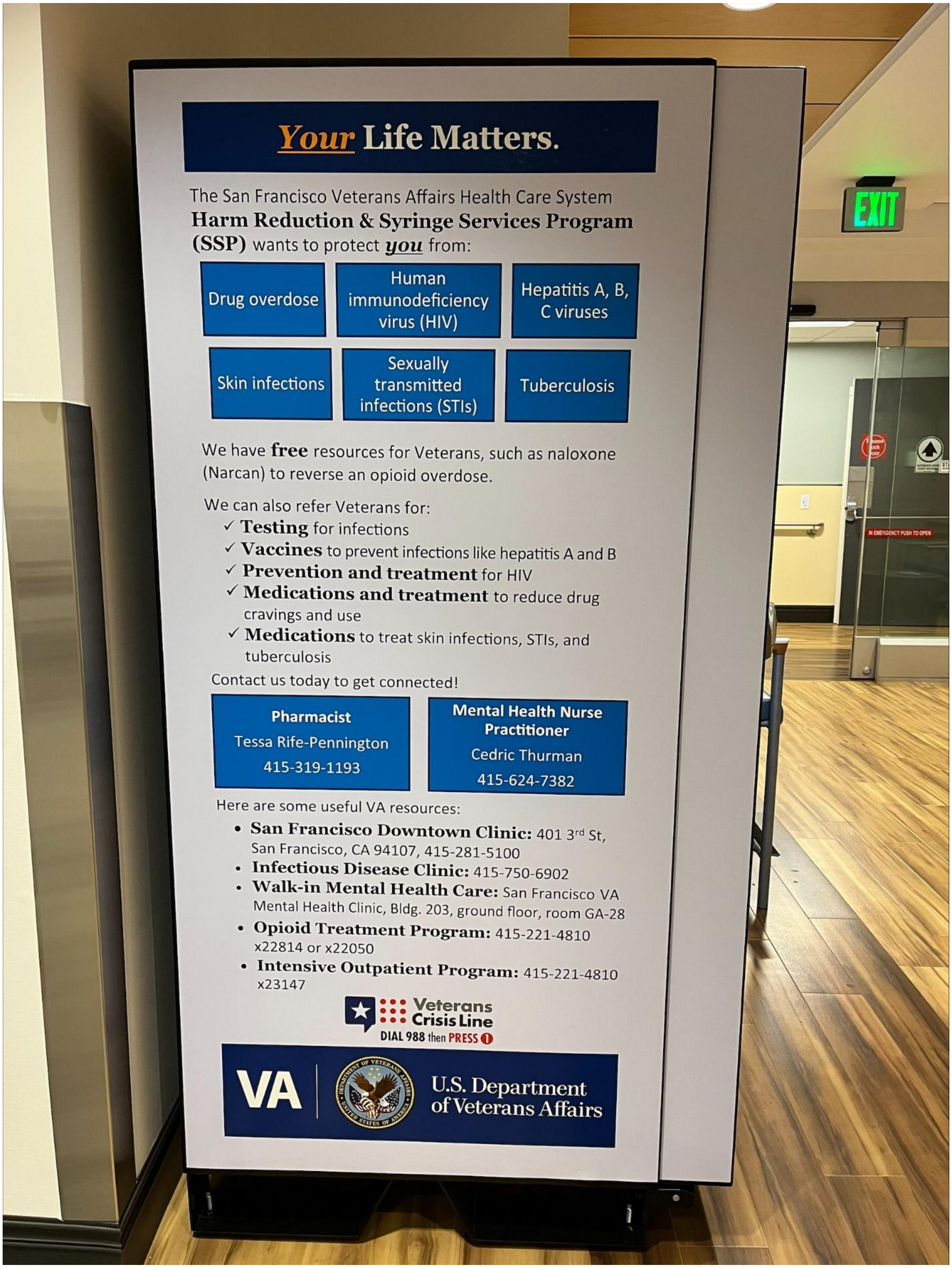
Your Life Matters.
The San Francisco Veterans Affairs Health Care System
Harm Reduction & Syringe Services Program
(SSP) wants to protect you from:
Drug overdose
Human immunodeficiency virus (HIV)
Hepatitis A, B, C viruses
Skin infections
Sexually transmitted infections (STIs)
Tuberculosis
We have free resources for Veterans, such as naloxone (Narcan) to reverse an opioid overdose.
We can also refer Veterans for:
✓ Testing for infections
✓ Vaccines to prevent infections like hepatitis A and B
✓ Prevention and treatment for HIV
✓ Medications and treatment to reduce drug cravings and use
✓ Medications to treat skin infections, STIs, and tuberculosis
Contact us today to get connected!
Pharmacist
Tessa Rife-Pennington
415-319-1193
Mental Health Nurse Practitioner
Cedric Thurman
415-624-7382
Here are some useful VA resources:
• San Francisco Downtown Clinic: 401 3rd St, San Francisco, CA 94107, 415-281-5100
• Infectious Disease Clinic: 415-750-6902
• Walk-in Mental Health Care: San Francisco VA Mental Health Clinic, Bldg. 203, ground floor, room GA-28
• Opioid Treatment Program: 415-221-4810 x22814 or x22050
• Intensive Outpatient Program: 415-221-4810 x23147
Veterans Crisis Line
DIAL 988 then PRESS 1
VA
U.S. Department of Veterans Affairs
EXIT

Appendix 2. Bar Code Wallet Card

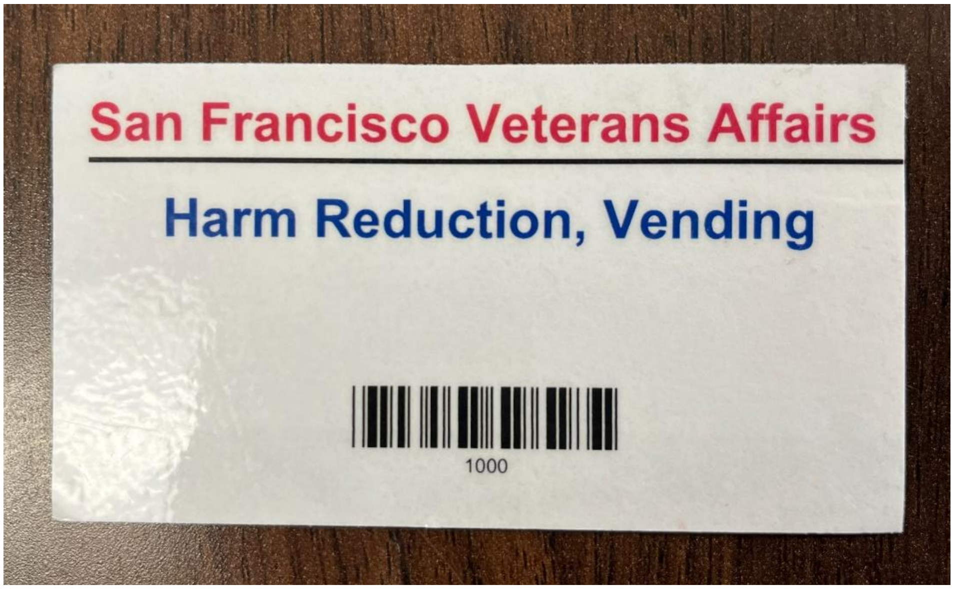

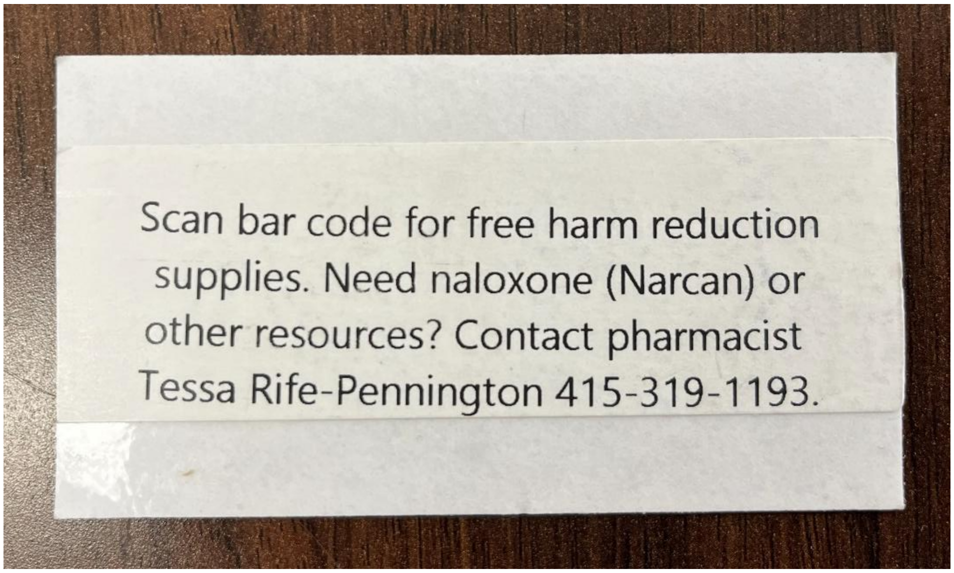

Appendix 3. Harm Reduction Vending Machine Contents.

Body lotion 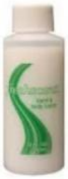	Deodorant 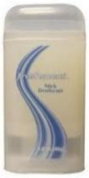	Alcohol-free mouthwash 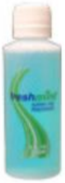	Lip balm 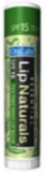
Hand sanitizer 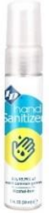	Sunscreen 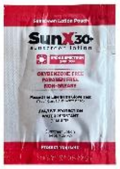	Hygiene kit 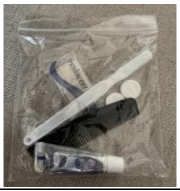	Wound care kit 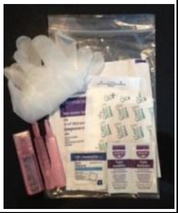
Alcohol swabs 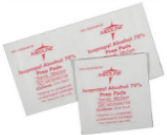	1-quart sharps container 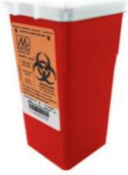	Personal sized sharps container 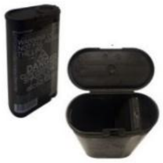	Latex-free tourniquet 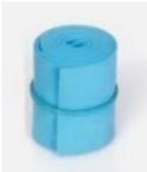
External (penile) latex condoms 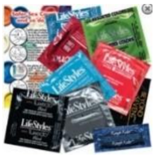	XL external (penile) latex condoms 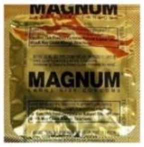	Water-based lubricant 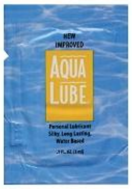	Ascorbic acid (vitamin C) powder 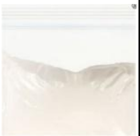
Sterile water vial. 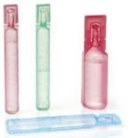	Test strips to check drugs for fentanyl 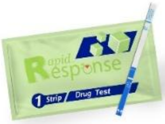	Test strips to check drugs for xylazine (AKA Tranq) 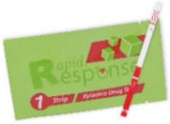	Cooker with a cotton pellet 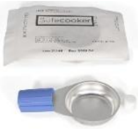
Rubber mouthpiece 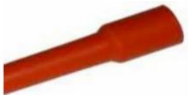	Safer snorting kit 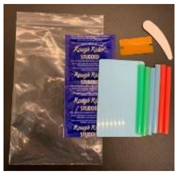	Safer rectal drug use kit (AKA boofing, booty bumping) 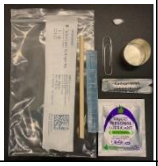	**<u>27G</u>** 16mm 1mL syringes 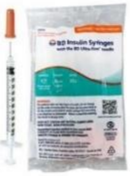
12mm 1mL syringes 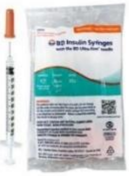	12mm 1mL syringes 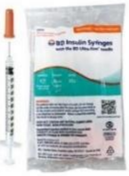	12mm 1mL syringes 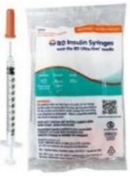	8mm 1mL syringes 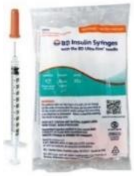

Appendix 4. Dispensing Limits

Item	Allowed Per Day
27G insulin syringe	1 bag (10 syringes)
28G insulin syringe	1 bag (10 syringes)
29G insulin syringe	1 bag (10 syringes)
30G insulin syringe	1 bag (10 syringes)
31G insulin syringe	1 bag (10 syringes)
Alcohol swabs	1 bag (10 swabs)
Ascorbic acid (vitamin C) powder	1 bag
Cooker with cotton filter	6 cookers with filters
Safer rectal use kit	2 kits
Sterile water vial	6 vials
Wound care kit	1 kit

Item	Allowed Per Week
1 quart sharps container	1 container
Alcohol-free hand sanitizer	1 bottle
Aqua lube	2 bags (20 single-use packets)
Deodorant	1 bottle
Fentanyl test strips	1 bag (10 test strips)
Hygiene kit	1 kits
Latex condoms	2 bags (20 condoms)
Lip balm	1 tube
Lotion	1 bottle
Mouthwash	1 bottle
Personal size sharps container	1 container
Rubber mouthpiece	1 mouthpiece
Xylazine test strips	1 bag (10 test strips)
XL latex condoms	2 bags (20 condoms)
Safer snorting kit	7 kits<br>**starting 7/1/24, changed from 1/day
Sunscreen	7 bags (3 single-use packets)<br>**starting 7/1/24, changed from 1/day
Tourniquet	7 tourniquets<br>**starting 7/1/24, changed from 1/day
